# Selection of lncRNAs That Influence the Prognosis of Osteosarcoma Based on Copy Number Variation Data

**DOI:** 10.1155/2022/8024979

**Published:** 2022-03-26

**Authors:** Jian Zhang, Chi Huang, Guiqi Zhu, Guanyi He, Wenbo Xu, Jianming Li, Dong Wang, Kecheng Han, Zilong Shen, Jianyu Liu

**Affiliations:** ^1^Department of Orthopedics, The Second Affiliated Hospital of Harbin Medical University, Harbin, 150001 Heilongjiang, China; ^2^Department of Rheumatology and Immunology, Zaozhuang Municipal Hospital, 277102 Shandong, China; ^3^Department of Orthopedics, The Fifth Hospital of Harbin, Harbin, 150040 Heilongjiang, China

## Abstract

Osteosarcoma is the most common primary malignancy in the musculoskeletal system. It is reported that copy number variation- (CNV-) derived lncRNAs contribute to the progression of osteosarcoma. However, whether CNV-derived lncRNAs affect the prognosis of osteosarcoma remains unclear. Here, we obtained osteosarcoma-related CNV data and gene expression profiles from The Cancer Genome Atlas (TCGA) database. CNV landscape analysis indicated that copy number amplification of lncRNAs was more frequent than deletion in osteosarcoma samples. Thirty-four CNV-lncRNAs with DNA-CNV frequencies greater than 30% and their corresponding 294 mRNAs were identified. Gene Ontology (GO) and Kyoto Encyclopedia of Gene and Genome (KEGG) pathway enrichment analyses revealed that these mRNAs were mainly enriched in olfaction, olfactory receptor activity, and olfactory transduction processes. Furthermore, we predicted that a total of 23 genes were *cis*-regulated by 16 CNV-lncRNAs, while 30 transcription factors (TFs) were *trans*-regulated by 5 CNV-lncRNAs. Through *t*-tests, univariate Cox regression analysis, and the least absolute shrinkage and selection operator (LASSO), we constructed a CNV-related risk model including 3 lncRNAs (AC129492.1, PSMB1, and AC037459.4). The Kaplan-Meier (K-M) curves indicated that patients with high-risk scores showed poor prognoses. The areas under the receiver operating characteristic (ROC) curves (AUC) for predicting 3-, 5-, and 7-year overall survival (OS) were greater than 0.7, showing a satisfactory predictive efficiency. Gene set enrichment analysis (GSEA) revealed that the prognostic signature was intimately linked to skeletal system development, immune regulation, and inflammatory response. Collectively, our study developed a novel 3-CNV-lncRNA prognostic signature that would provide theoretical guidance for the clinical prognostic management of osteosarcoma.

## 1. Introduction

Osteosarcoma is the most widespread primary bone tumor [1], occurring mainly at the age of 15-19 years, and poses a serious threat to the health of patients. Prior to the 1970s, patients with osteosarcoma were primarily treated only with surgery, with an event-free survival (EFS) rate of approximately 20% [2]. Since the late 1970s, the treatment of osteosarcoma patients has tended to surgery combined with chemotherapy [3]. Currently, the international standard perioperative regimen of osteosarcoma patients includes several chemotherapeutic drugs, such as high-dose methotrexate and doxorubicin and cisplatin (MAP), but other treatments have a little clinical meaningful impact [4–6]. Pleasingly, the introduction of multiagent chemotherapy several decades ago has improved the 5-year EFS for localized high-grade osteosarcoma from less than 20% to around 60% [7]. However, patient survival and clinical status have not improved significantly in recent decades, and the treatment of surgery combined with polychemotherapy remains insufficient [8]. Therefore, identifying biomarkers is important to improve the clinical status of patients with osteosarcoma.

Long noncoding RNAs (lncRNAs) are one of the major regulatory factors of gene expression and play an important role in cancers, including osteosarcoma [9]. Studies have shown that lncRNAs can affect the proliferation, migration, invasion, apoptosis, and other biological processes of osteosarcoma cells and can predict the recurrence and prognosis of osteosarcoma [10–14]. For example, lncRNA SNHG4 can promote cell proliferation and migration by sponging miR-377-3p in osteosarcoma [10]. lncRNA SNHG3 is involved in the invasion and migration of osteosarcoma by regulating the miRNA-151a-3p/RAB22A axis [11]. Moreover, lncRNA SNHG4 is associated with tumor growth and poor prognosis of osteosarcoma patients [13]. Therefore, lncRNAs may be promising targets for the new advanced treatment of osteosarcoma.

Copy number variant (CNV) is a region of the genome that varies in integer copy numbers, including DNA sequence amplifications and deletions, and can drive rapid adaptive evolution and progression of cancers [15]. Increasing evidence has revealed that systematic screening of CNV can identify new biomarkers to improve diagnosis and targets for therapeutic interventions of osteosarcoma [16]. Interestingly, recent studies have shown that both the deletion and amplification for transcribing genes of lncRNAs can affect the occurrence and development of cancer to a certain extent [17]. However, the relationship between CNV-derived lncRNAs and the prognosis of osteosarcoma has rarely been studied.

In the present study, we firstly identified lncRNAs related to CNV in osteosarcoma. Next, we investigated the functions and regulatory mechanisms of CNV-lncRNAs. Moreover, we established a CNV-lncRNA risk signature for predicting the prognosis of osteosarcoma. Finally, we also developed a nomogram based on the risk score and clinical features for more accurately predicting the prognosis of osteosarcoma. Collectively, this study might provide insight into the mechanism of CNV in osteosarcoma and contribute to improving the treatment of osteosarcoma.

## 2. Materials and Methods

### 2.1. Data Acquisition

Transcriptome and CNV profiling data of 87 osteosarcoma samples were downloaded from TCGA database (https://portal.gdc.cancer.gov) to screen CNV-lncRNAs. A total of 77 samples with survival and clinical information were used to construct a prognostic risk signature. The clinical information of these 77 osteosarcoma samples is displayed in Table 1.

### 2.2. Identification of CNV-lncRNAs

The mean value of segment was usually employed to evaluate the CNV of DNA fragments and identify a CNV gain or a CNV loss type. CNV-derived lncRNAs in 87 osteosarcoma samples were identified using the following thresholds: segment mean > 2.78 for the gain and segment mean < 1.72 for the loss. Next, CNV profiles of 87 osteosarcoma samples were selected by manipulating Genomic Identification of Significant Targets in Cancer (GISTIC) software (https://www.genepattern.org/) to further visualize copy deletion and amplification of lncRNAs. False discovery rate (FDR) value < 0.05 was considered significant importance. Moreover, lncRNAs with DNA-CNV alteration rates greater than 30% were first screened in the osteosarcoma CNV data. Namely, individuals with gain + loss accounted for more than 30% of all individuals. Furthermore, the above-harvested lncRNAs with expression profiles in osteosarcoma samples were termed as CNV-lncRNAs.

### 2.3. Investigation of the Regulatory Mechanism for CNV-lncRNAs

The lncRNAs mainly affect mRNA expression through *cis*- and *trans*-regulation. The mRNAs that met the following criteria were considered *cis*-acting genes: the mRNA site was located within 300 kb either upstream or downstream of the lncRNAs. Briefly, the specific positions of lncRNAs and genes are obtained using the annotation file (A) of the human genome downloaded from the NCBI database, and a threshold of ∣distance | <300 kb was set for filtering; then, the relative positions of lncRNAs and genes were calculated. The algorithm for the distance is specified as follows: if the lncRNA is all contained in the gene or the gene is all contained in the lncRNA, then the distance is 0; if the lncRNA is upstream of the gene, the calculation of “lncRNA start position (lnc.S)-gene start position (gene.S)” and “lncRNA end position (lnc.E)-gene end position (gene.E)” should be performed, and the distance of the lncRNA should be the lowest absolute value. The relative position of lncRNA should be negative. If the lncRNA is downstream of the gene, the “lnc.S-gene.S” and “lnc.E-gene.E” algorithms are executed, again taking the smallest absolute value as the distance, in which case the relative position of the lncRNA should be positive.

For *trans*-acting genes, we screened the TFs of lncRNAs obtained from the Human Transcription Factor Database (http://humantfs.ccbr.utoronto.ca/index.php) to construct a lncRNA-TF network. Specifically, the lncRNA-mRNA relationship pairs were obtained by calculating the correlation between mRNA and lncRNA, filtered at *P* < 0.05 and ∣cor | >0.7; then, the TF list obtained from the Human Transcription Factor Database was utilized as a basis to identify the overlapping genes with the harvested mRNAs; finally, the lncRNA-overlapping gene relationship pairs were collated and the lncRNA-TF network was constructed by Cytoscape software.

### 2.4. Functional Enrichment Analysis

Functional enrichment analysis of coding genes associated with CNV-lncRNAs was performed to reveal potential molecular mechanisms of lncRNAs. The psych package in R was applied to detect the relationship between lncRNAs and mRNAs to further screen the mRNAs mostly associated with osteosarcoma under the selection criteria of *P* value < 0.05 and ∣cor | >0.7. The potential functions and pathways of these harvested mRNAs were explored by GO and KEGG analyses using the clusterProfiler package in R. The value of *P* less than 0.05 was accepted as significant enrichment.

### 2.5. Construction of a Prognostic Risk Signature

The osteosarcoma samples with survival information were classified into a training set and a testing set randomly at a rate of 5 : 5. The clinical information of the training and testing sets is illustrated in Table 2.

In the training set, we utilized univariate Cox regression analysis to screen lncRNAs related to the survival of osteosarcoma with the cut-off value = 0.2. LASSO analysis was used to identify the candidate prognostic lncRNAs using the glmnet package in R to construct a risk signature for osteosarcoma. The risk score of each osteosarcoma patient sample was calculated using the following formula: Risk score = *e*^sum(each gene's expression levels^×^corresponding coefficient)^/*e*^sum(each gene's mean expression levels^×^corresponding coefficient)^

We divided osteosarcoma patients into a high-risk group and a low-risk group based on the median risk score. K-M survival analysis was performed to assess the prognostic difference of the two risk groups with the survival package in R. The predictive power of the risk signature was detected by the ROC analysis using the R package survivalROC. The above results were also validated in the testing set.

### 2.6. Construction of a Nomogram

According to the clinical information of 77 osteosarcoma patients, univariate and multivariate Cox regression analyses were employed to identify independent factors from clinical characteristics (metastatic, gender, and age) and risk scores for osteosarcoma. The R package of rms was used to establish a nomogram based on all independent factors to predict the survival probability of 1, 3, and 5 years for osteosarcoma patients. Moreover, the calibration curve and proportional hazard assumption were separately plotted and calculated to access the predictive efficiency of the nomogram.

### 2.7. GSEA

GSEA was conducted to investigate the biological functions of prognostic lncRNAs using the clusterProfiler R package based on the GO and KEGG analyses.

### 2.8. Statistical Analysis

The correlation between the risk score and clinical characteristics was evaluated by the *t*-test. The RCircos package was used to visualize the distribution of lncRNA copy number amplification and deletion in the genome. In this study, *P* < 0.05 was regarded as a statistically significant difference.

## 3. Results

### 3.1. Analysis of CNV for lncRNAs in TCGA Database

**Figure 1:**
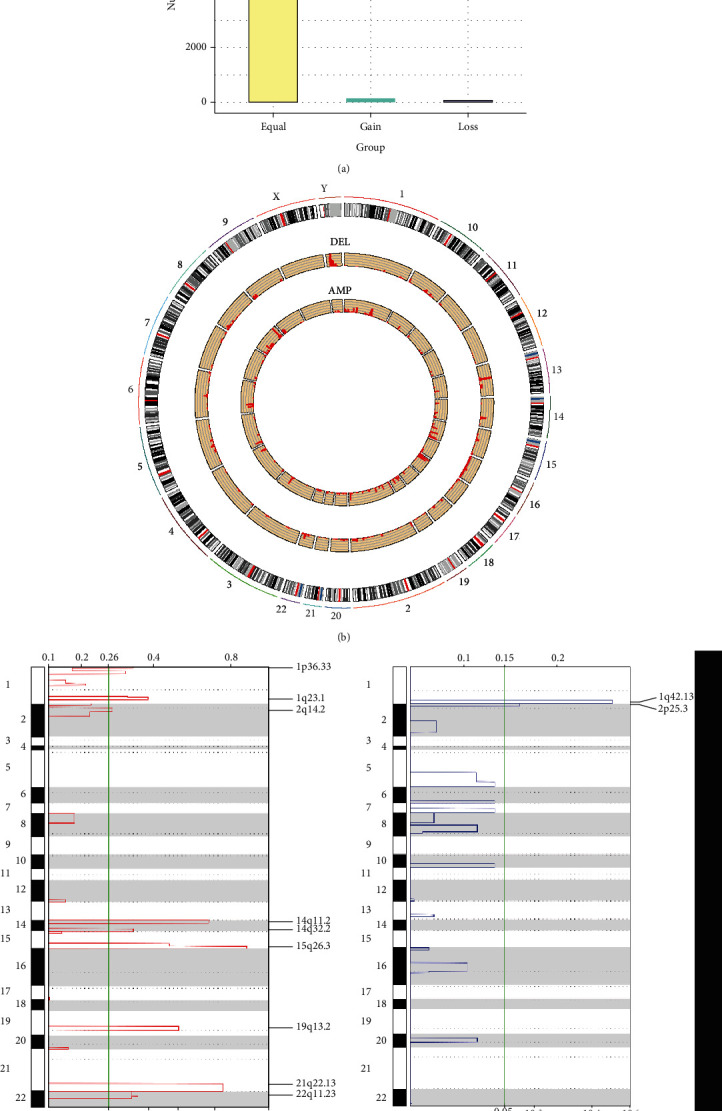
Copy number profile of lncRNAs in osteosarcoma: (a) copy number of lncRNAs in osteosarcoma samples; (b) lncRNA copy loss and copy amplification proportion distribution in the genome; (c) the lncRNAs located in the focal CNA peaks are OS-related. False discovery rates (*q* values) and scores from GISTIC 2.0 for alterations (*x*-axis) are plotted against genome positions (*y*-axis); dotted lines indicate the centromeres. The amplifications and deletions of lncRNA genes are also shown. The green line represents 0.05 *q* value cut-off point that determines significance.

CNV is a common form of structural change in the whole genome that is reported to be an important contributor to tumor development [18]. In our study, we firstly assessed the spectrum of CNV on the whole genome. The results revealed a small portion of CNV of lncRNAs in osteosarcoma samples, and the frequency of copy number amplifications was greater than deletions (Figures 1(a) and 1(b)). Moreover, the GISTIC algorithm was selected to detect the frequently changed regions in the osteosarcoma genome. As shown in Figure 1(c), the focal amplification events were mostly concentrated on 1p36.33, 1q23.1, 2q14.2, 14q11.23, 14q32.2, 15q26.3, 19p13.2, 21q22.13, and 22q11.23, while the focal deletion events were mainly concentrated on 1q42.13 and 2p25.3. In summary, the above results highlighted the impact of copy number amplification of lncRNAs in osteosarcoma.

### 3.2. Analysis of CNV-lncRNAs in TCGA-Osteosarcoma

To screen CNV-lncRNAs that were closely associated with osteosarcoma, we first screened 61 lncRNAs with >30% CNV alteration rate based on TCGA-osteosarcoma DNA-CNV data (Supplementary Table 1); meanwhile, 34 lncRNAs (Supplementary Table 2) with detectable expression levels were identified from the above 61 lncRNAs according to TCGA-osteosarcoma expression profiles, which were termed as CNV-lncRNAs.

**Figure 2:**
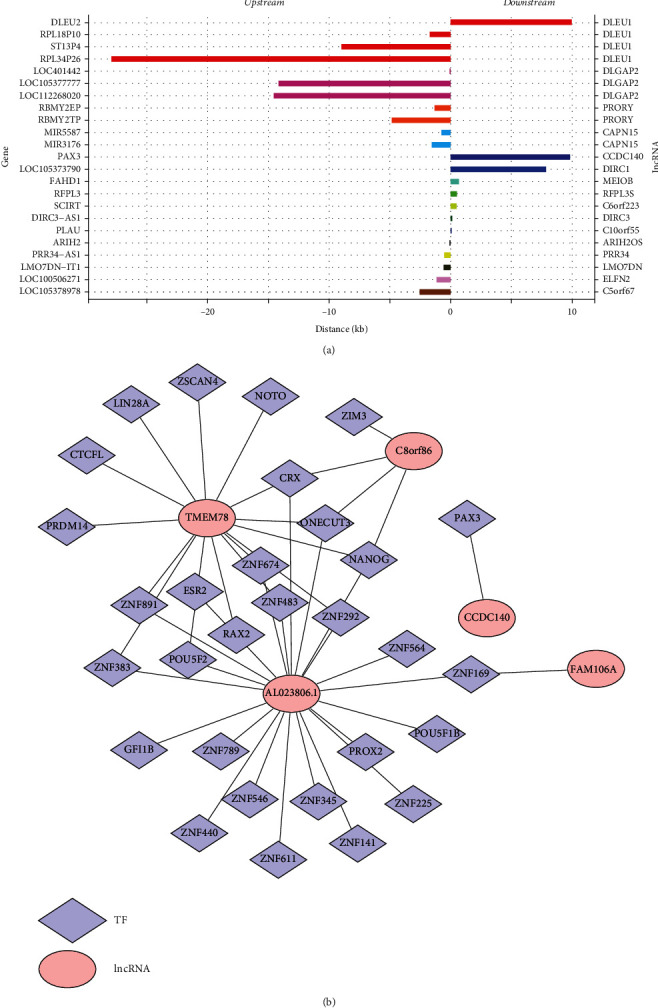
Prediction of *cis*- and *trans*-acting genes of harvested lncRNAs: (a) *cis*-regulated mRNAs by CNV-lncRNAs; (b) lncRNA-TF network constructed by lncRNAs and their *trans*-acting target genes.

Next, we preliminarily explored the regulatory mechanisms of CNV-lncRNAs. In terms of *cis*-fashion, 23 mRNAs were found to be regulated by 16 CNV-lncRNAs, and the detailed information is illustrated in Figure 2(a) and Supplementary Table 3. For example, the expression levels of RPL18L10, ST13P4, and RPL34P26 were *cis*-regulated by DELU1. Additionally, 30 TFs were predicted to be the *trans*-regulated genes of these 5 lncRNAs, containing AL023806.1, TMEM78, FAM106A, C8orf86, and CCDC140. Then, these lncRNAs and their respective *trans*-acting target genes were used to establish a lncRNA-TF network (Figure 2(b)).

Besides, to explore the potential functions of these CNV-lncRNAs, we calculated the correlation of 34 CNV-lncRNAs with 19,513 protein-coding genes detected in osteosarcoma (significant correlation threshold set to ∣cor | >0.7 and *P* < 0.05) using the psych package in R. A total of 294 coding genes significantly associated with 10 CNV-lncRNAs (AL023806.1, TMEM78, C3orf36, CCDC140, C8orf86, FAM106A, CABIN1, PSMB1, CAPN15, and C10orf55) were screened (Supplementary Table 4), which were utilized to perform functional enrichment analysis (Supplementary Table 5). GO analysis showed that these genes were mainly involved in 3 BP entries and 1 MF entry (Supplementary Figure 1A), which were notably correlated with olfactory sensory perception. KEGG enrichment analysis suggested that these genes were markedly related to the olfactory transduction pathway (Supplementary Figure 1B). Therefore, these 10 CNV-lncRNAs may be closely associated with olfactory sensory perception-related functions, but the relationship between olfactory function and osteosarcoma is currently unexplored.

### 3.3. Prognostic Value of lncRNAs in Osteosarcoma

**Figure 3:**
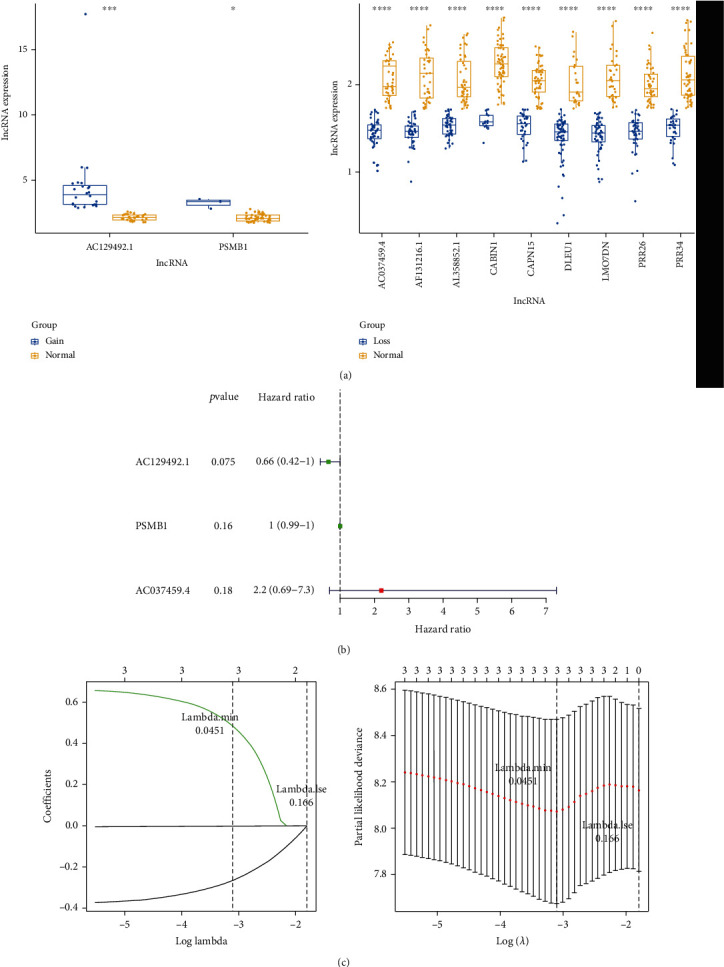
Screening of prognostic lncRNAs in osteosarcoma: (a) lncRNA expression in CNV-gain and CNV-loss groups; (b) forest map based on the univariate Cox regression analysis of lncRNAs; (c) fivefold cross-validation for tuning parameter selection in the LASSO model. Partial likelihood deviance is plotted against log (*λ*), where *λ* is the tuning parameter. Dotted vertical lines were drawn at the optimal values by minimum criteria and 1-s.e. criteria (left). LASSO coefficient profiles of the 3 lncRNAs associated with the prognosis of osteosarcoma in the univariate Cox regression analysis (right).

**Figure 4:**
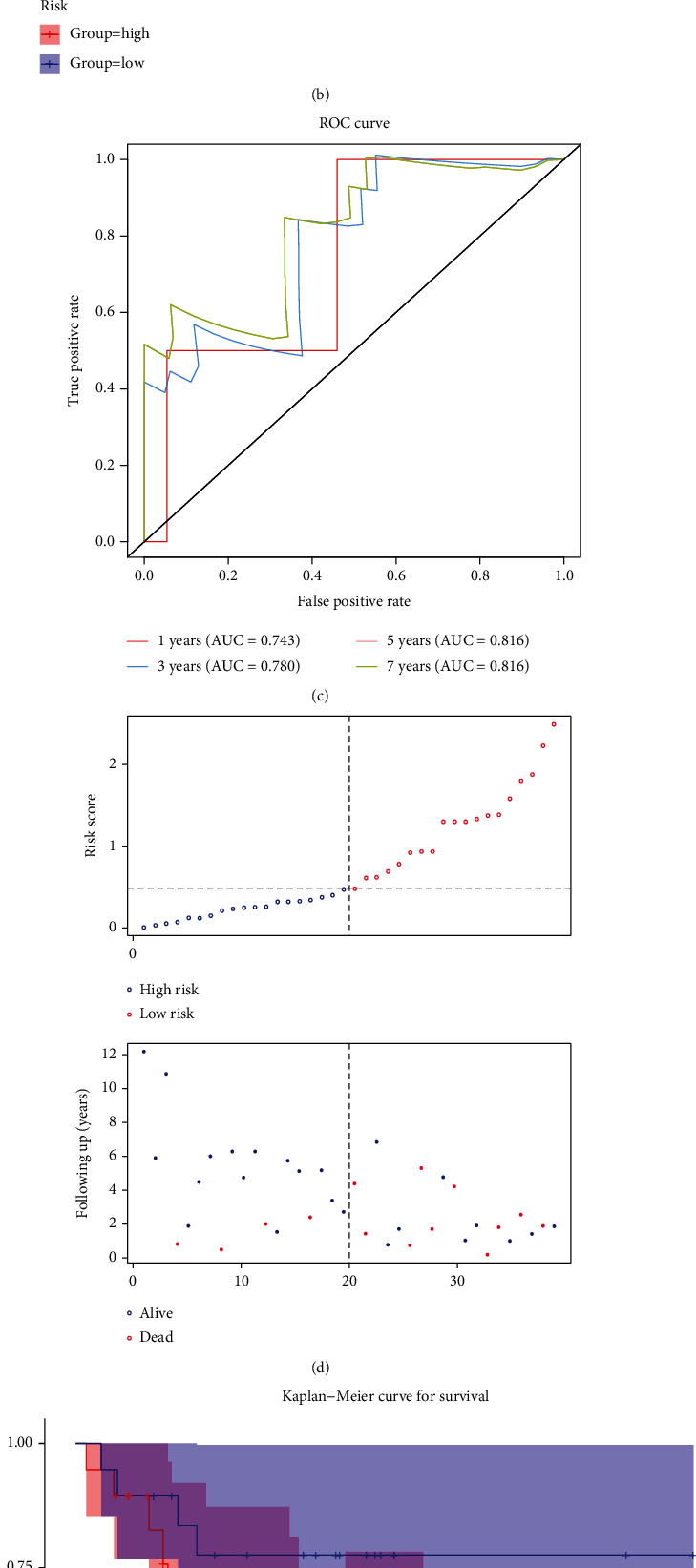
Generation of risk signature. (a) The risk curve of each sample was reordered by risk score (up).The scatter plot showed the overall survival status of osteosarcoma patients in the training cohort (middle). The heat map showed the expression of prognostic genes in the training cohort (bottom). (b) The survival curve showed the different overall survival statuses between high- and low-risk patients. (c) Receiver operating characteristic curves of prognostic signature in the training cohort. (d) Risk score model plot including risk score ranking, survival status, and heat map in the testing cohort. (e) Kaplan-Meier plot of the risk score model in the testing cohort. (f) ROC curves for 1-, 3-, 5-, and 7-year survival rates from the risk score model in the testing cohort.

Among 34 lncRNAs, the expression levels of AC129492.1 and PSMB1 were increased in the CNV-gain groups, while AC037459.4, AF131216.1, AL358852.1, CABIN1, CAPN15, DLEU1, LMO7DN, PRR26, and PRR34 were all lowly expressed in the CNV-loss group (Figure 3(a)). Univariate Cox regression analysis was performed on the lncRNAs to select the lncRNAs associated with the prognosis of the training set-osteosarcoma. AC129492.1 (*P* = 0.075, HR = 0.66, 95%CI = 0.42 − 1), PSMB1 (*P* = 0.16, HR = 1, 95%CI = 0.99 − 1), and AC037459.4 (*P* = 0.18, HR = 2.2, 95%CI = 0.69 − 7.3) were identified (Figure 3(b)). Next, AC129492.1, PSMB1, and AC037459.4 were reserved and used to construct a prognostic risk signature for osteosarcoma based on LASSO analysis (Figure 3(c) and Table 3). Moreover, osteosarcoma patients in the training set were divided into the high-risk and low-risk groups according to the median risk score (Figure 4(a)). The K-M survival curve demonstrated that the two risk groups exhibited different overall survival (OS), and the high-risk score was related to a poor prognosis (*P* = 0.04, Figure 4(b)). The AUC value of 1, 3, 5, and 7 years reached 0.743, 0.780, 0.816, and 0.816, respectively (Figure 4(c)). The distribution of patients with osteosarcoma under different clinical characteristics between the high- and low-risk groups in the training set is available in Supplementary Table 6.

The predictive accuracy of the risk signature was also verified in the testing set. The detailed information of risk score distribution and survival status is illustrated in Figure 4(d). The patients with high-risk scores presented shorter OS than those with low-risk scores (*P* = 0.0074, Figure 4(e)). Consistent with the above results, the risk signature had positive performance for predicting osteosarcoma (1-year AUC = 0.431, 3-year AUC = 0.774, 5-year AUC = 0.844, and 7-year AUC = 0.954; Figure 4(f)). The distribution of patients with osteosarcoma under different clinical characteristics between the high- and low-risk groups in the testing set is available in Supplementary Table 7.

### 3.4. Investigation of the Relationship between Risk Score and Clinical Characteristics in Osteosarcoma

**Figure 5:**
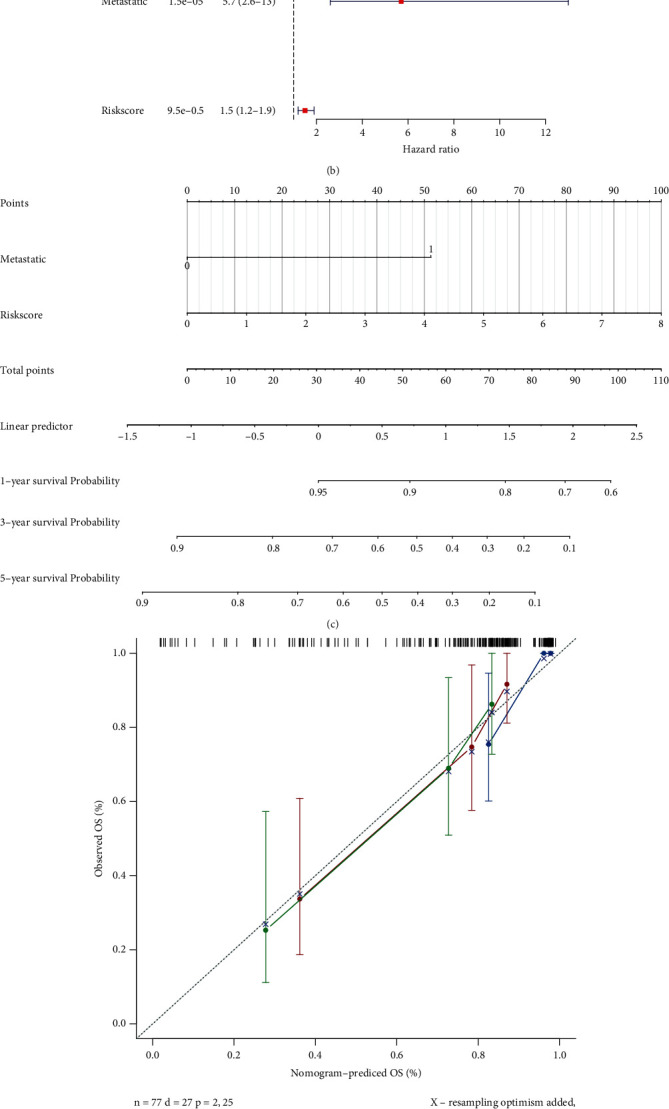
Nomograms based on the independent prognostic factors in patients with osteosarcoma: (a) univariate Cox analysis of overall survival-related variables; (b) multivariate Cox analysis of overall survival-related variables; (c) nomogram to predict 1-, 3-, and 5-year overall survival probability by integrating the risk score and clinicopathologic risk factors; (d) plot depicting the calibration of the nomogram in terms of the agreement between predicted and observed outcomes. Nomogram performance is shown by the plot relative to the dotted line, which represents perfect prediction. (e) Proportional hazard assumption revealed that the nomogram had good predictive efficiency.

In 77 osteosarcoma samples with survival information, metastasis and the risk score were identified as independent factors by the univariate and multivariate Cox regression analyses (*P* < 0.05, Figures 5(a) and 5(b)). Based on the selected independent factors, a nomogram was generated to predict the survival probability of 1, 3, and 5 years that showed a similar trend with the ideal curve (Figures 5(c) and 5(d)). Moreover, proportional hazard assumption also showed that the nomogram had good predictive efficiency (Figure 5(e)). To summarize, the constructed nomogram had a better predictive power for predicting the survival probability.

### 3.5. Functional Enrichment of Risk Signature

**Figure 6:**
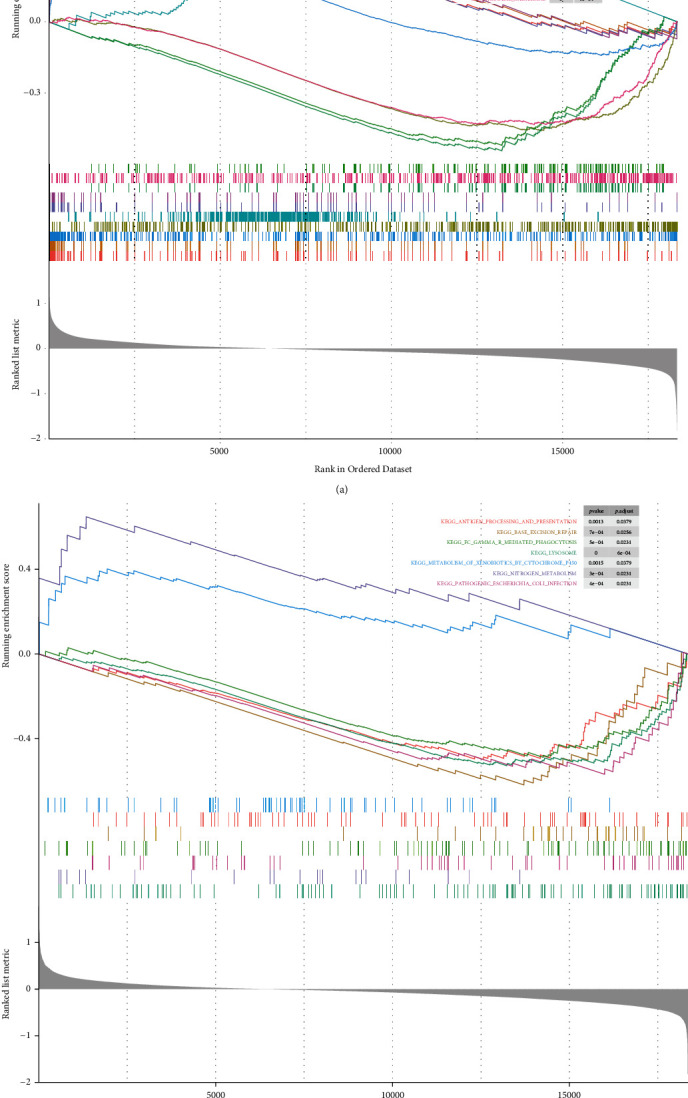
Identification of the functional pathways of the risk score: (a) enriched gene sets annotated by the GO collection between the high- and low-risk groups in the whole TCGA cohort; (b) enrichment plots showing the KEGG gene sets between the high- and low-risk groups in the whole TCGA cohort. Enrichment score (ES): a positive ES indicates gene set enrichment at the top of the ranked list; a negative ES indicates gene set enrichment at the bottom of the ranked list. The ranking metric measures a gene's correlation with a phenotype.

GO analysis (Figure 6(a); Supplementary Table 8) indicated that skeletal system-related terms were significantly enriched in the high-risk group (NES > 1), such as “REPLACEMENT OSSIFICATION”, “BIOMINERALIZATION”, “BONE MINERALIZATION”, “BONE MORPHOGENESIS”, “OSTEOBLAST DIFFERENTIATION”, “BONE DEVELOPMENT”, and “SKELETAL SYSTEM MORPHOGENESIS”; meanwhile, they may also be involved in cartilage (“ENDOCHONDRAL BONE MORPHOGENESIS” and “CARTILAGE DEVELOPMENT”), muscle (“SKELETAL MUSCLE ADAPTATION”, “SKELETAL MUSCLE CONTRACTION”, “MUSCLE FIBER DEVELOPMENT”, “MUSCLE CONTRACTION”, etc.)-related processes and “CONNECTIVE TISSUE DEVELOPMENT”. In contrast, the low-risk group was closely associated with mitochondria-related functions (“MITOCHONDRIAL TRANSLATION”, “MITOCHONDRIAL TRANSLATIONAL TERMINATION”, “MITOCHONDRIAL GENE EXPRESSION”, etc.), immune regulation (“REGULATION OF MYELOID LEUKOCYTE MEDIATED IMMUNITY”), and inflammatory response (“REGULATION OF ANTIGEN PROCESSING AND PRESENTATION” and “ANTIGEN PROCESSING AND PRESENTATION”); in addition, immune cell physiological regulation (“REGULATION OF LEUKOCYTE DEGRANULATION”, “MYELOID LEUKOCYTE DIFFERENTIATION”, and “REGULATION OF LEUKOCYTE DIFFERENTIATION”), tumor necrosis factor regulation (“NEGATIVE REGULATION OF TUMOR NECROSIS FACTOR” and “TUMOR NECROSIS FACTOR MEDIATED SIGNALING PATHWAY”), and “APOPTOTIC CELL CLEARANCE” were also significantly enriched (all NES < −1). KEGG demonstrated that the high-risk group was markedly associated with “NITROGEN METABOLISM” and “METABOLISM OF XENOBIOTICS BY CYTOCHROME P450”; “LYSOSOME”, “PATHOGENIC ESCHERICHIA COLI INFECTION”, “BASE EXCISION REPAIR”, “ANTIGEN PROCESSING AND PRESENTATION”, and “FC GAMMA R MEDIATED PHAGOCYTOSIS” were considerably enriched in the low-risk group (Figure 6(b); Supplementary Table 9). This evidence suggests that the poorer prognosis of patients in the high-risk group may be related to abnormalities of the skeletal system.

## 4. Discussion

Osteosarcoma is the most common primary malignant bone tumor, occurring most frequently in children and adolescents (median age 18 years), and the 5-year survival rate of osteosarcoma patients remains poor [19]. Casual inspection of published data indicates that survival of osteosarcoma patients has not improved further since the 1970s and that chemotherapy drugs used today appear to be exactly similar to those used 30 years ago [20]. Therefore, lacking specific and sensitive biomarkers to predict the prognosis of osteosarcoma patients is still an urgent issue to be addressed. Increasing evidence has suggested that lncRNAs play an important role in the occurrence and progression of osteosarcoma by affecting several biological processes and may be related to prognosis and recurrence of osteosarcoma [21, 22]. In addition, recent studies have revealed that CNV-lncRNAs can serve as predictions for cancer patients [23]. However, the role of CNV-lncRNAs in osteosarcoma has not been clarified.

In this study, we firstly identified 34 CNV-lncRNAs by analyzing the transcriptome and CNV profiling. Subsequently, a total of 10 DE-lncRNAs between the CNV and control groups were screened. Moreover, using univariate and LASSO Cox regression analyses, we constructed a prognostic risk signature based on AC129492.1, PSMB1, and AC037459.4 for osteosarcoma.

To our knowledge, lncRNA PSMB1 is the first to be found in cancers, while AC129492.1 and AC037459.4 have been reported in other cancers. For instance, AC129492.1 is associated with the prognosis of hepatocellular carcinoma and may regulate immune response in hepatocellular carcinoma [24]. Moreover, Yin et al. also found that AC129492.1 can affect the prognosis in colon cancer and may be involved in the regulation of genome instability [25]. Thus, our research further revealed that AC129492.1 may play a key role in cancers. On the other hand, consistent with our results, AC037459.4 has been suggested to be related to a mutation in hepatocellular carcinoma [26]. Thus, we speculated that AC129492.1, PSMB1, and AC037459.4 may have great significance in osteosarcoma. However, their specific roles need to be further studied in the future.

Notably, we also found that the risk signature was relevant to bone mineralization and nitrogen metabolism. It has been suggested that poor mineralization can promote the adhesion of cancer cells, resulting in the development of cancer [27]. Moreover, lung cancer cells also can inhibit bone mineralization, which may be related to the bone metastasis of lung cancer [28]. It is widely known that nitrogen is essential for the growth of cancer and immune cells [29–31]. Recent research showed that leucine and branched-chain amino acid metabolism can provide the energy for osteosarcoma cells and keep it growing [32]. Thus, we speculated that AC129492.1, PSMB1, and AC037459.4 may be involved in the progression of osteosarcoma by regulating bone mineralization and nitrogen metabolism.

There have been many studies on the prognosis of osteosarcoma. Clinically, a set of PET/CT indicators can provide valuable information for the prognosis of patients with osteosarcoma, and serum miRNA can be used as a biomarker for the prognosis of osteosarcoma [33, 34]. Moreover, many immune-related genes are associated with the prognosis of osteosarcoma in the study of immune microenvironment and tumor microenvironment [35–39]. In addition, some scholars have screened lncRNA and finally constructed the prognosis and recurrence risk signature of osteosarcoma [22, 40]. Compared to these risk signatures, we were the first time to construct a risk signature based on CNV-lncRNAs, and our risk signature showed good predictive power for predicting 5- and 7-year OS. Moreover, another innovation in this study was that we constructed a nomogram for predicting 1-, 3-, and 5-year OS of osteosarcoma patients by integrating the risk score and other clinical features (metastasis), and calibration plots suggested that the nomogram has efficient performance.

## 5. Conclusion

This paper mainly studied the significance of CNV-lncRNA in the prognosis of osteosarcoma by bioinformatics methods and firstly concluded that AC129492.1, PSMB1, and AC037459.4 could be used as prognostic markers of osteosarcoma, which may provide theoretical basis and reference value for the study and prognosis of osteosarcoma in the field of copy number and noncoding RNA. However, the sample size included in this study was small, and some important clinical data could not be obtained from the database. Moreover, the molecular mechanisms of AC129492.1, PSMB1, and AC037459.4 remained murky. Therefore, in future work, we will further study the roles and molecular mechanisms of AC129492.1, PSMB1, and AC037459.4 in the pathogenesis and development of osteosarcoma, to better promote the improvement of the asymptomatic survival rate of osteosarcoma patients.

## Figures and Tables

**Table 1:** Clinicopathological characteristics of TCGA-osteosarcoma patients.

Characteristics	Groups	Patients (*N* = 77)	
		No.	%
Age (days)	Mean	5516.62	
	Range	2177-11828	
	<5000	32	41.56
	≥5000	45	58.44
Gender	Male	35	45.45
	Female	42	54.55
Metastatic	Yes	21	27.27
	No	56	72.73

**Table 2:** Clinical information for training and testing sets.

Variable		Training set (*n* = 39)	Testing set (*n* = 38)	*P* value
Age (days, mean (range))		5563.39 (2177-10205)	5471.05 (1299-11828)	0.8233
Gender	Male	19	16	0.7235
	Female	20	22	
Metastatic	Yes	9	12	0.5608
	No	30	26	

**Table 3:** Regression coefficient of the prognostic lncRNAs.

lncRNA name	Coefficient
AC129492.1	-0.263486134
PSMB1	-0.002824893
AC037459.4	0.48461758

## Data Availability

The data used to support the findings of this study are included within the article.

## Supplementary Materials

Supplementary Materials Supplementary Figure 1: GO function annotation and KEGG pathway enrichment analyses. (A) The bubble plots for GO function enrichment (biological process). The color of the dot stands for the different *P* values, and the size of the dot reflects the number of target genes enriched in the corresponding pathway. (B) The bar diagrams for KEGG pathways. The *y*-axis represents the pathways, and the *x*-axis represents enriched gene numbers, and the color means adjusted *P* value. Supplementary Table 1: lncRNAs with >30% CNV alteration rate. Supplementary Table 2: expression profiles of 34 CNV-lncRNAs in TCGA database. Supplementary Table 3: *cis*-regulatory relationships of 23 mRNAs and 16 CNV-lncRNAs. Supplementary Table 4: results of Pearson analysis of coding genes significantly associated with CNV-lncRNAs. Supplementary Table 5: results of GO and KEGG enrichment analysis of 294 coding genes significantly associated with CNV-lncRNAs. Supplementary Table 6: clinical information of high- and low-risk groups in the training set. Supplementary Table 7: clinical information for the high- and low-risk groups in the test set. Supplementary Table 8: GO enrichment analysis of risk score-related genes. Supplementary Table 9: KEGG enrichment analysis of risk score-related genes.
