# Supplementary material for: Selection of lncRNAs That Influence the Prognosis of Osteosarcoma Based on Copy Number Variation Data
Source: J Oncol. 2022 Mar 26;2022:8024979. doi: 10.1155/2022/8024979 (PMC8976607; doi:10.1155/2022/8024979)
Supplement: Supplementary Materials — Supplementary Figure 1: GO function annotation and KEGG pathway enrichment analyses. (A) The bubble plots for GO function enrichment (biological process). The color of the dot stands for the different P values, and the size of the dot reflects the number of target genes enriched in the corresponding pathway. (B) The bar diagrams for KEGG pathways. The y-axis represents the pathways, and the x-axis represents enriched gene numbers, and the color means adjusted P value. Supplementary Table 1: lncRNAs with >30% CNV alteration rate. Supplementary Table 2: expression profiles of 34 CNV-lncRNAs in TCGA database. Supplementary Table 3: cis-regulatory relationships of 23 mRNAs and 16 CNV-lncRNAs. Supplementary Table 4: results of Pearson analysis of coding genes significantly associated with CNV-lncRNAs. Supplementary Table 5: results of GO and KEGG enrichment analysis of 294 coding genes significantly associated with CNV-lncRNAs. Supplementary Table 6: clinical information of high- and low-risk groups in the training set. Supplementary Table 7: clinical information for the high- and low-risk groups in the test set. Supplementary Table 8: GO enrichment analysis of risk score-related genes. Supplementary Table 9: KEGG enrichment analysis of risk score-related genes. [file 8024979.f1.zip › 8024979.f10.pdf]

ID	Descriptior	setSize	enrichmen	NES	pvalue	p.adjust	qvalues	rank
KEGG_LYS(	LYSOSOMI	121	-0.5276	-1.75662	3.27E-06	0.000598	0.000506	5127
KEGG_NITF	NITROGEN	23	0.647965	2.101929	0.000338	0.023083	0.019518	1338
KEGG_PAT	PATHOGEI	55	-0.56805	-1.74549	0.000441	0.023083	0.019518	2361
KEGG_FC_(	FC GAMM/	95	-0.48811	-1.59035	0.000505	0.023083	0.019518	4095
KEGG_BAS	BASE EXCI!	33	-0.61846	-1.74029	0.0007	0.025619	0.021662	4640
KEGG_ANT	ANTIGEN F	80	-0.49798	-1.59796	0.001322	0.037922	0.032065	5786
KEGG_MET	METABOLI	68	0.40174	1.672714	0.001451	0.037922	0.032065	1934

leading_edcore_enrichment
tags=52%, 285362/2799/2720/4126/3074/2548/1213/10717/2990/4669/4074/4668/3425/1522/872
tags=26%, 761/1373/2744/760/759/767
tags=35%, 10552/3059/81873/23643/387/84617/71/3875/7454/25/203068/10095/10381/84790/10
tags=43%, 653361/5604/3635/2214/6199/10163/1785/5594/2934/4082/5293/5879/7409/8853/651
tags=70%, 4913/4350/143/54107/79661/7515/4595/5425/3978/27343/56655/8930/57804/328/496
tags=41%, 3134/3115/3312/8302/3305/3123/3119/3326/3304/567/6891/925/8625/811/5641/6890
tags=15%, 2944/1545/2949/130/119391/2947/1559/126/125/10941

[illegible]2/57192/1174/3482/53/5641/23163/1514/10053/1200/535/1201/26088/1211/2629/6272/8943/15[illegible]

[illegible]383/60/7280/7846/929
[illegible]08/10552/1398/8612/81873/208/27040/23533/5580/23396/8936/1794/7454/1072/7408/2212/529
[illegible]8/10038/142/5111/2237/5426/10039/27301/5424
[illegible]/3310/3823/3108/3821/1514/972/926/1508/3135/6892/3105/3106/920/3303/10437/3107/3822

[illegible]8/4864/527/537/9179/8907/2517/4125/84572/533/3073/6556/4758/9374/23457/245972/5476/91

6/3055/273/10095/2213/5880/56848/3984/2209/8877/1759

14/162/10577/51606/3373/1509/1497/3916/51172/10312/7805/23659/8218/968/54

LYSOSOME
NITROGEN METABOLISM
PATHOGENIC ESCHERICHIA COLI INFECTION
FC GAMMA R MEDIATED PHAGOCYTOSIS
BASE EXCISION REPAIR
ANTIGEN PROCESSING AND PRESENTATION
METABOLISM OF XENOBIOTICS BY CYTOCHROME P450
