# Supplementary material for: Selection of lncRNAs That Influence the Prognosis of Osteosarcoma Based on Copy Number Variation Data
Source: J Oncol. 2022 Mar 26;2022:8024979. doi: 10.1155/2022/8024979 (PMC8976607; doi:10.1155/2022/8024979)
Supplement: Supplementary Materials — Supplementary Figure 1: GO function annotation and KEGG pathway enrichment analyses. (A) The bubble plots for GO function enrichment (biological process). The color of the dot stands for the different P values, and the size of the dot reflects the number of target genes enriched in the corresponding pathway. (B) The bar diagrams for KEGG pathways. The y-axis represents the pathways, and the x-axis represents enriched gene numbers, and the color means adjusted P value. Supplementary Table 1: lncRNAs with >30% CNV alteration rate. Supplementary Table 2: expression profiles of 34 CNV-lncRNAs in TCGA database. Supplementary Table 3: cis-regulatory relationships of 23 mRNAs and 16 CNV-lncRNAs. Supplementary Table 4: results of Pearson analysis of coding genes significantly associated with CNV-lncRNAs. Supplementary Table 5: results of GO and KEGG enrichment analysis of 294 coding genes significantly associated with CNV-lncRNAs. Supplementary Table 6: clinical information of high- and low-risk groups in the training set. Supplementary Table 7: clinical information for the high- and low-risk groups in the test set. Supplementary Table 8: GO enrichment analysis of risk score-related genes. Supplementary Table 9: KEGG enrichment analysis of risk score-related genes. [file 8024979.f1.zip › 8024979.f2.pdf]

lncRNA name	alteration rate
DLEU1	0.738636364
AC129492.1	0.659090909
LMO7DN	0.636363636
FAM106A	0.613636364
AL133475.1	0.590909091
C10orf55	0.579545455
AL365273.1	0.545454545
DLGAP2	0.534090909
AC037459.4	0.534090909
AC008522.1	0.534090909
AF131216.1	0.534090909
AC087392.1	0.522727273
AL358852.1	0.511363636
AL023806.1	0.511363636
AC013286.1	0.511363636
PRR26	0.5
ARIH2OS	0.488636364
PSMB1	0.477272727
PRORY	0.477272727
AC009121.2	0.477272727
AC108925.1	0.465909091
C9orf92	0.454545455
DIRC3	0.454545455
C8orf86	0.454545455
AC009950.1	0.443181818
AC113404.1	0.443181818
CCDC140	0.431818182
AC242988.1	0.431818182
AC008641.1	0.431818182
TMEM78	0.431818182
AC092718.1	0.420454545
AL513412.1	0.420454545
AC007906.1	0.409090909
PATL2	0.409090909
AC022405.1	0.409090909
CAPN15	0.409090909
AC073657.1	0.409090909
AC009065.4	0.397727273
AC008914.1	0.397727273
C5orf67	0.397727273
MEIOB	0.386363636
DIRC1	0.386363636
PRR34	0.386363636
AC245100.1	0.386363636
AC005042.1	0.375
AC010184.1	0.375
MAPK4	0.375
C3orf36	0.375
AC234582.1	0.375
AC138028.1	0.363636364

AC092384.1	0.363636364
AC004158.1	0.352272727
AL008723.1	0.352272727
AL590235.1	0.352272727
AL365181.2	0.329545455
RFPL3S	0.329545455
C6orf223	0.318181818
AC104389.2	0.318181818
ELFN2	0.306818182
AC126407.1	0.306818182
CABIN1	0.306818182
C11orf40	0.295454545
AP000866.1	0.295454545
AC008686.1	0.295454545
AC087762.1	0.284090909
AC012363.2	0.284090909
SLC22A18AS	0.284090909
AC006449.2	0.284090909
L34079.2	0.272727273
C8orf44	0.272727273
AC104534.3	0.272727273
SPATA8	0.272727273
DSCR4	0.272727273
DSCR8	0.272727273
AC073333.1	0.272727273
AC092159.1	0.261363636
FAM182B	0.261363636
PRNT	0.261363636
TMEM99	0.25
AL133335.1	0.238636364
AC100821.2	0.238636364
AL645728.1	0.227272727
AJ239318.1	0.227272727
AC005480.1	0.227272727
HCG27	0.227272727
LINC01620	0.215909091
FAM218A	0.215909091
AL049794.1	0.215909091
C14orf177	0.215909091
AC104532.2	0.215909091
AC079907.1	0.215909091
CT62	0.215909091
AC006538.4	0.215909091
AC123512.1	0.204545455
AC016549.1	0.204545455
CLLU1	0.193181818
AL589743.1	0.193181818
BLACE	0.193181818
KIFC1	0.193181818
C7orf77	0.193181818
C14orf178	0.193181818

AP005242.1	0.193181818
MDS2	0.181818182
AC073130.2	0.181818182
MAP3K14	0.181818182
HHLA3	0.181818182
AL357140.1	0.170454545
AC110615.1	0.170454545
DNAH10OS	0.170454545
AL162426.1	0.159090909
C2orf91	0.136363636
AC022210.1	0.125
AC107081.1	0.125
AC253572.1	0.113636364
AL445183.1	0.102272727
AL356289.1	0.090909091
