# Supplementary material for: Selection of lncRNAs That Influence the Prognosis of Osteosarcoma Based on Copy Number Variation Data
Source: J Oncol. 2022 Mar 26;2022:8024979. doi: 10.1155/2022/8024979 (PMC8976607; doi:10.1155/2022/8024979)
Supplement: Supplementary Materials — Supplementary Figure 1: GO function annotation and KEGG pathway enrichment analyses. (A) The bubble plots for GO function enrichment (biological process). The color of the dot stands for the different P values, and the size of the dot reflects the number of target genes enriched in the corresponding pathway. (B) The bar diagrams for KEGG pathways. The y-axis represents the pathways, and the x-axis represents enriched gene numbers, and the color means adjusted P value. Supplementary Table 1: lncRNAs with >30% CNV alteration rate. Supplementary Table 2: expression profiles of 34 CNV-lncRNAs in TCGA database. Supplementary Table 3: cis-regulatory relationships of 23 mRNAs and 16 CNV-lncRNAs. Supplementary Table 4: results of Pearson analysis of coding genes significantly associated with CNV-lncRNAs. Supplementary Table 5: results of GO and KEGG enrichment analysis of 294 coding genes significantly associated with CNV-lncRNAs. Supplementary Table 6: clinical information of high- and low-risk groups in the training set. Supplementary Table 7: clinical information for the high- and low-risk groups in the test set. Supplementary Table 8: GO enrichment analysis of risk score-related genes. Supplementary Table 9: KEGG enrichment analysis of risk score-related genes. [file 8024979.f1.zip › 8024979.f3.pdf]

id	TARGET-4	TARGET-4	TARGET-4	TARGET-4	TARGET-4	TARGET-4	TARGET-4	TARGET-4
AC008522.	1.71892	0.335566	0	0.828392	1.58269	0.419983	0.164684	0.060098
AC037459.	0.617221	0.439826	0	0.593198	0.43767	0.106242	0	0
AC113404.	0	0	0	0.121101	0	1.37776	0.174766	0
AC129492.	0.232519	0.197583	3.33682	0.411481	0.602055	2.19823	0.290953	6.86839
AC245100.	2.71327	7.94	6.10463	5.50717	8.05602	7.0094	4.99282	4.90221
AF131216.	0.115766	0.101114	0.504831	0.198153	0.116024	0.052706	0.140659	0.19268
AL023806.	11.3213	1.28531	3.0815	0.801796	0.772759	1.6639	8.5673	1.62302
AL358852.	0.044406	0.217107	0.707423	0.424202	0.314803	0.303095	0.389083	0.170064
ARIH2OS	5.77773	1.6373	2.63859	0.523678	0.76069	0.587493	4.25596	1.38465
C10orf55	0.938014	0.209797	0.53643	0.093351	0.524592	0.959159	1.35814	1.37731
C3orf36	0.031566	0.223765	0.032854	0.031833	0	0.077241	0	0.032973
C5orf67	0.435815	0	0	0	0	0	0.158322	0
C6orf223	2.03378	0.163744	0.056798	0.225304	0.974143	0.958469	0.122095	1.0031
C8orf86	6.08317	0.105128	0.496574	0	0.064644	0.177446	2.22804	0.28909
C9orf92	0	0.119546	0.528869	1.07902	0	0.758918	1.96947	0.643231
CABIN1	15.8044	20.8121	26.8774	16.8729	19.3461	27.9294	26.8914	15.344
CAPN15	21.4773	18.6625	31.4453	20.8598	23.7516	22.4149	30.7111	12.3519
CCDC140	0.193403	0	0.385429	0.849878	0.393241	0	0.025635	0.444587
DIRC1	0	0	0	0.164318	0.065846	0.15172	0	0.064459
DIRC3	0.05374	0.763562	0.435233	1.51488	0.64615	0.812734	0.195559	0.169386
DLEU1	31.9421	14.7942	1.87005	7.82137	14.6991	12.892	20.572	7.62567
DLGAP2	0.417598	0.179074	7.75814	0.061103	0.282868	0.171362	0.717818	0.07837
ELFN2	0.015068	0.295889	0.436879	0.105751	0.013254	0.031654	0.251116	0.000849
FAM106A	8.11148	0.64102	0.331627	1.20041	2.62836	0.929236	2.79142	0.434927
LMO7DN	0.647788	2.22641	0.649483	0.430262	0.051822	2.44557	1.51596	0.133671
MAPK4	0.882665	0.128554	4.24491	0.123446	0.058848	0.009082	0.347239	0.00556
MEIOB	1.33817	1.69523	7.04472	0.119542	0.474869	0.424421	0.505792	0.110082
PATL2	3.33529	0.695627	3.39233	1.38159	1.70269	1.57245	6.03835	0.769175
PRORY	4.50549	0.057336	0.179842	0.039548	0.224113	0.080694	1.94495	0.346558
PRR26	0.932755	0.099509	0.253885	0.052229	0.155675	0.425222	0.998743	0.221384
PRR34	0.737171	0.328875	0.316717	0.064936	0.152366	0.50435	1.51739	0.174656
PSMB1	498.894	217.44	161.98	277.007	319.987	162.634	86.2265	114.699
RFPL3S	0.419874	0.121646	2.44663	1.4702	1.31011	0.390368	5.17019	0.201662
TMEM78	4.52201	0.694117	0.485897	0.111216	0.656332	0.370293	2.98763	0.137079

TARGET-4	TARGET-4	TARGET-4	TARGET-4	TARGET-4	TARGET-4	TARGET-4	TARGET-4	TARGET-4
0.19831	0.031268	0.303071	0.354053	1.20311	0	0.481632	0.057889	1.95933
0.356726	0.580305	0.739201	0	0	0.459758	0.667276	0.15922	0.151406
0.098144	0.226341	0.154523	0	0.524512	0.174547	0	0.274172	0
2.70499	3.02211	2.67291	5.30332	2.36748	2.40028	7.8694	1.39263	0.369611
3.15409	6.29398	2.80625	5.8187	4.01551	5.16179	9.89105	6.77772	4.62684
0.294323	0.099489	0.172341	0	0	0	0.232136	0.045897	0.184053
2.13639	5.01891	3.74249	1.89489	5.37427	1.77277	0.923219	3.22202	1.71714
0.769044	0	0.168299	0.552328	0.113204	0.146198	0.552202	0.579577	0.284832
2.24359	2.4587	0.836769	1.06568	2.49615	1.19853	1.68804	1.89748	1.30599
0.755362	1.08491	0.414541	0.495607	1.22186	0.24724	0.168344	0.697241	0.421538
0.211481	0.040374	0	0.20492	0.124098	0	0.307427	0	0.608334
0.022116	0	0	0.294776	1.07748	0.25817	0	0	0.224431
0.787355	1.52238	0.096881	0.267213	0.053003	0.092511	0.487489	6.34402	0.183599
0.226148	1.69638	0.282333	0.095599	0.820556	0.181462	0.143628	0.123368	0.118896
0	0	0	0.509114	0	0	0	0.20182	0
41.1397	37.0913	33.283	38.3498	49.9763	16.7462	24.692	54.5241	28.8712
29.3704	33.1839	32.8793	42.9428	25.5542	29.2688	33.7503	20.9628	21.8548
0.053543	0.287511	0	0	0.205294	0.073728	0.258305	0.16597	0.028845
0.224972	0	0	0.035191	0.134513	0.087359	0	0.058064	0.060716
1.67146	1.59469	0.470781	0.320499	0.224943	0.273468	0.464371	0.847455	0.094146
13.5814	13.6502	25.0566	4.32471	17.432	10.611	8.63701	13.7665	12.1142
0.24321	0	0.114822	0.349185	3.226	0.369221	0	0	0.160572
0.016494	0	0.001284	0.295469	0.002174	0.013036	0.060103	0.179535	0.032308
1.7093	5.28517	1.63858	1.52311	0.731212	3.00421	1.59127	2.53437	0.500098
0.347404	4.50024	4.11418	0.969746	3.01217	0.076772	1.9523	2.29752	2.53325
0.057145	0.072653	0	0	0	0.547423	0.02771	0	0.028047
1.6725	0.842711	0.191026	0.241195	0.535625	0.465133	0.374505	0.35973	0.503081
1.60664	2.65259	2.20366	2.20435	2.16732	0.611298	1.6891	2.49853	3.18434
0.14892	1.2239	0.317586	0.242217	0.451212	0.061941	0.038779	2.18833	0.796744
0.335749	3.63596	1.0702	0.451844	1.24245	0.355853	0.129535	0.207326	0.079085
0.360311	1.34327	0.194969	0.759113	1.55722	0.109134	0.108507	0.62375	0.480937
303.881	113.109	120.271	250.369	105.555	136.38	98.1925	339.534	276.854
2.35741	3.20674	8.09498	2.54187	1.85171	0.285508	0.170725	0.840723	1.47497
0.538181	1.60714	0.506592	0.312459	0.854824	0.636989	0.113896	0.174024	0.312905

TARGET-4(	TARGET-4(	TARGET-4(	TARGET-4(	TARGET-4(	TARGET-4(	TARGET-4(	TARGET-4(	TARGET-4(
0.066157	0	1.39142	0.81556	0.553209	0	0	0.09341	0
0.689257	0	0.099794	0	0.148392	0.497339	0.260737	0.608624	0.259494
0	0.265345	0	0.363487	0.461326	0.140635	0	0.094886	0
5.29216	1.1844	1.91656	2.30827	3.70829	3.03395	0.467198	0.643122	0.793966
4.43311	8.08629	3.89905	4.9269	10.4166	7.3571	4.33682	11.3105	1.96057
0.338693	0.189181	0.031714	0.273999	0.148423	0.068996	0.167918	0.23044	0.041074
0.730409	0.902759	3.31675	3.8463	1.28761	0.896199	0.711815	0.706713	0.490595
0.160823	0.037835	0.213418	0.397447	0.085627	0.693045	0.451023	0.734201	0.140698
1.00507	1.76931	1.94503	2.68148	2.53368	0.747529	0.537742	1.20604	0.699646
0.219213	0.65899	2.12604	1.40893	0.242595	0.234227	0.430202	0.297595	0.148259
0.319339	0	2.68855	0.144831	0.057705	0.096272	0.372889	0.806121	0
0	0	0.224207	0	0.048183	0	0.11744	0.239287	0
0.344451	2.43394	0.123308	0.56938	0.108263	0.152539	0.119309	0.274077	0.168374
0.073331	0.036298	0.461712	0.953031	0.151757	0.06925	0.054062	0.084778	0.251676
0	0	0.448413	0	0.085408	0.370908	0.128299	0	0
36.5857	15.7026	24.1965	47.633	37.5412	19.7821	16.8235	25.7711	8.45756
30.7311	14.7341	44.3485	51.9415	33.6196	22.2202	12.7679	13.333	20.683
0.180938	0.162803	0.128241	0.037925	0.1358	0	0.128222	0.037538	0
0.032921	0.314858	0.726193	0	0.024977	0.068919	0.021518	0	0
0.145051	5.45845	0.308885	0.453027	0.117206	1.81027	0.339869	0.397117	0.107541
6.07083	16.9493	18.2165	17.6058	6.32582	8.95112	4.46851	4.75213	10.0246
0.061494	0	0.037955	0.376174	0.181145	2.50938	0.155789	0.227434	0.01099
0.041921	0	0.069283	0.088451	0.940959	0.069264	0.027046	0.182035	0.05377
2.39593	0.957037	3.16791	2.60523	5.12035	0.542639	0.130939	0.172087	2.1059
0.75461	1.19887	1.21588	1.0399	0.665394	0.31906	0.432925	1.16828	0.235783
0.005309	0	0	0.008997	0.044591	0	0.050662	0.647463	0.025407
0	0	0.144748	0.390466	1.56285	0.097311	0.491453	0.29731	0.029487
0.713672	1.43784	2.7854	8.1463	1.03675	1.98616	0.452467	1.23131	0.369243
0.104247	0	0.287383	0.762038	0.493117	0.251334	0.079133	0.028604	0.109574
0.103652	0.003389	0.668696	1.27025	0.442021	0.097279	0.131176	0.407535	0.094539
0.319017	0.074026	0.724258	0.891861	0.516734	0.433219	0.307489	0.396397	0.162458
153.193	139.484	109.379	232.916	96.83	352.495	297.374	161.824	218.076
1.83648	3.09079	2.36434	3.05714	0.784927	1.11406	0.364872	0.460512	0.781619
0.079162	0.303452	0.828278	1.0242	0.209805	0.184188	0.150787	0.119646	0.339651

TARGET-4(	TARGET-4(	TARGET-4(	TARGET-4(	TARGET-4(	TARGET-4(	TARGET-4(	TARGET-4(	TARGET-4(
0.289143	0.293317	0.192186	1.01531	0.233205	1.54573	0	0	0.181714
0.223457	0.654054	0.26642	0	0.224741	0.185217	0	1.42026	0
0.066013	0.135486	0	0	0.136781	0	0	0.372648	0
1.0023	1.57833	3.04494	1.17247	0.968794	3.54424	4.20111	0.195821	0.942544
7.77046	10.295	6.32259	1.79884	8.64093	8.46927	13.0202	3.8481	3.23977
0.095774	0.078375	0.102605	0.204572	0.120089	0.192786	0.129047	0	0.059915
0.779844	0.79085	0.496256	0.435486	2.37328	4.98738	0.536642	0.898831	3.71242
0.25462	0.404078	0.116498	0.311109	0.333313	0.733833	0.212657	0.230177	0.244822
1.08845	1.12555	0.503173	1.09532	1.81436	3.06993	0.777945	0.951477	0.46514
0.266022	0.23897	0.139598	0.377394	0.10894	1.16661	0.192045	0.166659	0.246676
0.301778	0.073989	0.096917	0.011763	0.18881	0.241854	0	0.117802	0.829227
0.064058	0.344181	0	0	0.045159	0	0	0	0
0.080141	0.422414	1.36642	0.410262	0.011673	0.137456	0.124565	0.055159	0.657671
0.069954	0.022671	0.052758	0	0.357181	0.686086	0.188009	0.529631	0.229284
0.059616	0	0	0	0	0	2.1521	0	0
14.768	29.6995	22.721	17.5042	11.3336	63.988	19.7819	19.2311	9.92157
11.0394	12.1685	12.0364	8.53052	10.72	74.9894	15.9837	12.6926	29.3805
0.206125	0.453623	0.025132	0.010376	0.248174	0.036089	0.211138	0.711634	0.401902
0	0	0.05105	0.031297	0.166735	0.172743	0	0	0
0.364422	0.608719	0.228783	0.165403	1.3473	0.818722	1.24348	0.021472	0.686415
4.55955	8.38228	4.68824	6.49591	7.35994	14.2364	1.90303	10.4783	15.1129
0.115809	0.104488	0.004128	0	0.783612	0.182613	0	0.010688	0.007324
0.028054	0.004657	0.069835	0.020834	0.001753	0.025819	0.04441	0.045708	0.095892
0.263922	0.142126	0.575426	0.26374	1.48432	19.599	0.12324	0.636713	1.95352
0.708942	0.54958	0.393767	0.168947	2.57988	1.20854	0.173302	0.076115	1.70503
0.007747	0.020297	0	0	0.182033	0.135285	0.014827	0	1.53515
1.09471	3.89412	0.398177	0.051904	0.391788	0.611331	0.071909	0.097145	0.821783
1.56117	1.20641	1.34117	1.86443	1.26464	4.3206	1.0714	1.03964	0.319169
0.042931	0.106501	0.027084	0.048724	0.67341	0.685103	0.160348	0.128373	0.096329
0.060862	0.068247	0.074747	0.179656	0.230058	1.18112	0.04047	0.04733	0.175604
0.305109	0.714391	0.200715	0.414144	0.210504	0.977079	0.129206	0.14446	0.17714
149.679	239.06	160.145	98.4479	139.701	155.731	271.461	194.061	256.199
0.365795	1.41036	0.691497	0.570467	0.28042	15.2074	0.511426	1.22301	0.095658
0.099044	0.202144	0.046909	0.063112	0.753055	1.31034	0.162489	0.341959	0.59006

TARGET-4(	TARGET-4(	TARGET-4(	TARGET-4(	TARGET-4(	TARGET-4(	TARGET-4(	TARGET-4(	TARGET-4(
0.040902	0	0.050342	0	0	0.111229	0	0	0
0	0.4009	0	0	0	0.084279	0.250813	0.376487	0.227471
0	0	0	0	0.653654	0.280168	0.146939	0.079998	0
0	1.69449	0.648358	17.1393	3.59129	10.1986	6.23439	2.94236	2.22709
9.63032	2.73587	2.11201	3.51691	24.0366	4.11989	24.4006	6.64063	8.5288
0.088429	0.092622	0	0.285394	0.070694	0.107717	0.075061	0.290856	0.05249
1.47197	0.750652	0.404471	0.458132	1.3746	0.649966	1.13025	0.749967	0.892201
0.292667	0.366092	0.041205	0.780507	0.140751	0.139401	0.148038	0.135974	0.628815
1.95869	0.70905	0.248428	1.19106	1.07075	1.28628	0.802786	1.52678	0.748632
1.02805	0.237908	0.535533	0.379902	0.318124	0.21558	1.11166	0.73609	1.44684
0.51321	0	0.028327	0	0.136242	0.302409	0	0.08537	0.165782
0.310586	0.036782	0	0	0.291823	0.303314	0	0	0
0.085262	1.03485	0.068635	0.275833	0.940582	0.1136	0.34887	0.068607	0.186173
0.260233	0.18571	0.04163	0	0.052724	0.028219	0.076379	0.084616	0.053033
0	0	0	0	0.257319	0.385715	2.0078	0	0.913179
26.4619	22.851	7.60233	25.3737	26.2045	43.7005	34.0128	40.744	12.8535
13.7574	17.4276	3.14874	21.1232	48.8615	26.1432	23.8485	27.3204	17.6617
0.083538	0.013167	0.009844	0	0.077995	0.270824	0.022044	0	0
0	0.107923	0	0	0	0.05	0.02444	0.102197	0
0.458803	0.301985	0.184658	0.378182	0.071298	0.376045	1.89564	0.105935	0.022944
16.5207	6.5307	3.43132	19.9295	8.02344	5.69411	8.42357	7.20609	7.59935
0.017326	0	0.03552	1.13455	0.116819	0	0.056545	0.226558	0.192208
0.041081	0	0.008924	0.009954	0.026701	0.001701	0.095606	0	0
0.856305	0.623556	0.078394	0.239512	2.30993	0.423736	0.446018	0.532738	2.27986
0.290391	0.260517	0	0.131804	1.26705	0.317305	0.194343	0	0.95831
0.040819	0.058155	0.014164	0.038552	0.022176	0	0	0.025366	0
0.519727	0.121135	0.063428	0	0.192387	0	0.115856	4.34109	0.170836
2.4405	1.18659	0.547176	0.51005	1.0115	0.086796	0.813047	0.822579	0.807953
0.196167	0.13317	0.019536	0.033614	0.170119	0.039751	0.136064	0.172981	0.190042
0.014727	0.150634	0.054761	0.047436	0.358966	0.076836	0.150536	0.120395	0.18497
0.426192	0.464701	0.091454	0.331354	0.754496	0.143996	0.636371	0.192359	0.29145
167.714	314.52	49.279	322.239	279.9	166.458	159.209	310.997	316.175
2.87339	0.850348	0.049763	2.19877	0.337733	1.95992	0.563509	0.267329	0.422467
0.380347	0.209649	0.024666	0.090415	0.17481	0.041554	0.350446	0.119067	0.325563

TARGET-4(	TARGET-4(	TARGET-4(	TARGET-4(	TARGET-4(	TARGET-4(	TARGET-4(	TARGET-4(	TARGET-4(
0	0.364716	0.918761	0.041063	0.03364	0.070636	0.169299	0	0.016096
0.211512	0.891691	0.491898	0	0.632379	0.119901	1.37015	0.500575	0.118223
0.162991	0.143735	0.276443	0	0.164535	0.179988	0.206862	0.179689	0.519274
0	10.777	4.54519	1.12731	0.287824	1.08401	4.08056	3.72644	0
18.1141	1.38174	11.7343	3.54342	7.32592	0.809624	6.67753	3.88998	3.81812
0.08691	0.060388	0.238694	0.020624	0.281039	0.087073	0.382061	0.062371	0.306046
1.84586	1.91587	0.851368	0.272609	1.57353	0.448805	0.836251	0.81507	0.446444
0.811159	0.172906	0.552505	0.070209	0.793446	0.045881	0.466737	0.225008	0.331999
1.33262	1.90726	0.618289	0.247884	1.93177	0.45039	0.730756	0.925189	1.0533
2.37508	0.382344	0.362706	0.075196	0.350383	0.335188	0.361536	0.385761	0.759053
0.408179	0.215814	0.068147	0.032857	0	0	0.559584	0.151894	0.030216
0.064601	0	0.158235	0	0	0	0.368845	0	0
0.08185	0.043104	0.152322	0.054823	0.26375	0.622523	0.777373	0.520838	0.009849
0.240203	0.644533	0.138772	0.239748	0.42541	0.03883	0.152026	0.332851	0.096332
0	0	0.545605	0	0	0.737676	0.086857	0	0
42.2634	40.8929	41.4868	17.2879	18.4731	13.9775	50.8524	30.5829	17.4315
19.1822	18.5175	24.6141	27.4694	15.6819	9.70735	22.4377	21.0046	9.51731
0.10449	0.048329	0.045517	0.014792	0	0	0.253214	0.012712	0.127093
0.378106	0	0.056299	0	0	0.063611	0.022913	0	0
0.538136	0.242179	1.04505	0.498556	0.162476	0.336926	0.325643	0.243936	0.167433
5.82838	8.07598	5.88119	8.71267	13.0775	24.6476	4.32662	5.87856	7.61149
0.104536	0.023815	0.537763	0.081722	0.345765	0.002294	0.007479	0.052067	0.391669
0.110895	0.071411	0.001545	0.003001	0.070137	0.009353	0.056652	0.050211	0
0.642629	0.558247	0.249172	0.480801	1.54893	0.019757	0.417472	0.195383	0.098551
0.507663	1.6165	0.878825	0.133675	1.30075	0.074723	0.143287	0.050103	0.677153
0.127989	0.216849	0.033331	0	0.00902	0	0.013751	0.005986	0.011484
0.13086	0.566723	0.249759	0.439429	0.44974	0.337694	0.064326	0.038054	0.441463
2.59095	0.764286	1.99214	0.495741	1.03468	0.195568	0.243969	0.555785	1.52476
0.174605	0.280198	0.324105	0.079215	0.299334	0.01755	0.19272	0.157056	0.137944
0.162135	0.110217	0.212733	0.055135	0.239142	0.030175	0.042432	0.104689	1.19039
0.814127	0.63375	0.717982	0.160089	0.384132	0.091199	0.161244	1.06277	0.365858
319.745	107.079	202.324	484.003	227.595	237.481	98.0446	352.111	161.263
0.764757	2.57639	0.851543	0.431056	0.82505	0.216238	0.060111	0.489074	0.338524
0.302552	0.942848	0.228016	0.34868	0.567003	0.131724	0.225465	0.158128	0.087285

TARGET-4	TARGET-4	TARGET-4	TARGET-4	TARGET-4	TARGET-4	TARGET-4	TARGET-4	TARGET-4
0.230803	0.314487	0.874723	0.065235	0.177401	0	0.118241	0.38328	0.743574
0.060322	0	0	0.290602	0	0.034591	0	0.170812	0
0.117702	0.079089	0.119767	0.135556	0.081681	0.192175	0.140373	0.164966	0
0.963506	7.07965	5.29402	2.47898	3.93314	1.05686	0.828845	10.2601	0.000618
1.12572	17.1833	3.85555	16.6453	4.41006	4.3279	1.4734	19.0357	6.91547
0.101075	0.063473	0	0.26381	0.042376	0.105927	0.135767	0.459917	0.04236
2.56957	1.83386	3.18683	2.3539	1.81471	0.492932	0.46328	1.06602	1.2997
0.276229	0.053754	0.277023	0.895679	0.127081	0.212559	0.213376	0.323205	0.13859
0.612197	1.10921	1.51795	1.22325	1.81764	0.80896	0.9783	1.72514	0.672569
0.586482	0.551397	0.826181	1.08099	0.524204	0.396918	0.239178	0.675115	0.649309
0.378345	0.27202	0.354591	0.225318	0.051037	0.431604	0.388171	0.068289	0.125057
0	0.040049	0.37933	0.191279	0.127218	0.035227	0	0.383868	0
0.104066	0.101557	0.696451	0.087816	0.196396	0.016572	0.049267	0.518995	0.09407
0.340952	0.39163	0.216147	0.291515	0.339838	0.020054	0.084099	0.11379	0.172931
0.458918	0.319556	0.221116	2.00979	0.110344	0.332714	0.302681	0.10989	0.50968
19.9944	43.3432	63.1341	17.9813	60.0892	14.3118	14.159	44.0758	21.8932
9.26869	32.4464	22.0162	24.1313	19.6642	15.3119	12.6872	39.5941	31.956
0.072889	0.182548	0.480982	0.488665	0.149537	0.114085	0.068006	0.063957	0.056492
0.306199	0.027464	0.041452	0.500933	0	0	0	0	0
0.262534	1.72719	0.50042	0.494711	0.150044	1.27007	0.156512	0.481209	0.933573
6.28216	9.97175	10.4502	9.25027	10.2901	13.1747	5.51839	12.607	11.6814
0.260749	0.062143	0.090461	0.055435	0.049636	0.612736	0.006004	0.008231	0.00922
0.024264	0.187078	0.00253	0.077261	0.05419	0.001206	0.0734	0.091448	0.013705
0.472544	2.0684	2.3766	1.2946	0.441593	0.112456	0.077186	1.57321	2.77918
1.44505	0.899731	1.99255	0.485428	0.316257	0.277238	1.73421	0.023111	1.13202
0.130375	0.190684	0.028221	0.027024	0.022206	0.034792	0	2.25973	0.048177
0.188046	0.305535	0.274403	0.560761	0.040412	0.044565	0.18318	0.048168	0.068437
0.785972	0.636259	1.71807	4.06736	1.35055	1.62969	0.588643	1.10284	2.09633
0.104934	0.37443	30.1954	0.125919	2.16462	0.127175	0	0.174204	0.052524
0.108137	0.216505	0.266865	0.145651	0.083078	0.021193	0.019348	0.124815	0.290643
0.88501	0.383372	1.13531	0.748135	0.705439	0.207126	0.250757	0.422875	0.358991
139.887	225.011	99.2533	153.463	92.8591	361.863	283.362	317.459	168.864
0.889248	0.298253	3.37745	3.74089	1.67428	1.49718	2.05013	1.39921	0.332899
0.260897	0.438211	1.01409	0.473667	0.371944	0.052218	0.109331	0.284094	0.606722

TARGET-4	TARGET-4	TARGET-4	TARGET-4	TARGET-4	TARGET-4	TARGET-4	TARGET-4	TARGET-4
0.210926	0.26073	0	0.028604	0.066616	0.001058	0	0	0
0.056705	1.01984	0.131186	0.13239	0.120661	1.84357	0.648653	0.492803	0.200043
0.171269	0.705734	0.164418	0.079629	0	0	0.185827	0.580292	0.62441
1.16623	1.05301	5.18841	0.907135	1.697	2.07253	0.843682	5.9653	0.348814
3.99775	5.24207	15.761	7.99973	5.81041	10.65	4.38753	5.23143	15.5807
0.180706	0	0.166581	0.324637	0.108135	0.037009	0.104731	0.18516	0.084845
0.725875	24.3928	0.069631	1.23105	1.58631	1.10966	0.246955	0.788368	1.92601
0.030684	0.627103	0.025214	0.143717	0.176637	0.167484	0.112036	0.194767	0.237522
0.660337	3.11546	4.42689	1.15585	1.29132	1.43136	1.19849	0.970318	2.15817
0.12358	0.188899	0.050508	0.318754	0.466813	0.280497	0.304174	0.711986	2.17007
0.030711	0.193952	0	0.089342	0.043704	0.144122	0.107433	0	0
0	0.697542	0	0.089127	0	0.272774	0.562229	0	0
0.150575	0.351628	0.039714	0.026469	0.042772	0.282536	0.151205	0.064245	1.0764
0.019538	3.90337	0.055373	0.186708	0.258396	0.249176	0.22825	0.138878	0.093165
0	0	0	0.074348	0.874991	0	0.230719	0.276714	0
24.4278	14.7339	21.4608	19.4374	7.54962	15.2705	11.8165	33.9676	25.0503
16.8003	11.6135	10.9765	10.4519	13.5349	19.4776	7.88937	12.7286	23.5202
0	0	1.17243	0	0.040068	0	0	0	0.179013
0	0	0	0.024197	0	0.235942	0	0.402974	0.098154
0.009435	1.328	0.118779	2.49308	0.197535	0.283821	1.00197	3.25757	0.046025
4.64777	26.2938	13.2453	5.71878	8.01349	10.2272	6.21559	9.43419	12.0965
0.02393	1.25799	0.637829	0.029057	0.292887	0.413752	0.029911	0	0.005382
0.001462	0.714605	0.033439	0.002402	0.16877	0.193417	0.005356	0.004764	0.15081
0.720994	2.80349	0.153595	0.304945	0.834587	1.35615	0.245073	0.517837	0.649929
0	3.90627	0.191647	0.10451	0.03416	0.406974	0.189247	0.070812	0.082286
0.028598	0.945151	0	0.022908	0.041716	0.085435	0.007599	0.010195	0
1.01854	10.2982	0.875852	1.1729	3.37242	0.075289	0.055928	0.303834	0.093672
1.57961	7.0899	0.38898	4.69299	1.33961	0.542339	1.03685	0.996883	0.782906
0.099701	4.09005	0.049596	0.381091	0.136612	0.063566	0.100163	2.09528	0
1.29489	0.430399	0.029381	0.157898	0.119546	0.044701	0.03138	0.050188	0.377158
0.205709	0.904734	0.350724	0.440249	0.161883	0.213618	0.377017	0.628656	0.533144
403.757	157.043	269.331	187.954	183.982	145.366	178.534	130.278	129.541
1.39863	1.38679	0.474356	0.57877	0.54465	1.74437	0.83256	0.840689	1.66185
0.220204	8.85517	0.113652	0.368885	0.250469	0.448788	0.150885	0.139286	0.510571

TARGET-4(	TARGET-4(	TARGET-4(	TARGET-4(	TARGET-4(	TARGET-4(	TARGET-4(	TARGET-4(	TARGET-4(
0	0.339591	0.77592	0.371633	0.147298	0	0.594207	1.26568	0.034965
0	0	0.032848	0	0.03883	0.316839	0	0.211236	0.035756
0	0.351049	0	0	0.107649	0	0	0	0
1.52299	1.25391	0.666677	3.45614	0.998768	2.6833	1.19705	1.1716	0.888508
3.05708	2.64196	5.96854	16.1621	3.31288	9.52056	8.49852	2.42179	10.9822
0.352372	0.22622	0.315635	0.143668	0.060832	0.187755	0.21037	0.331719	0
0.900134	1.619	2.20411	0.921919	1.2467	0.710128	2.58913	2.41233	0.924066
0.132919	0.10007	0.940027	0.340775	0.219296	0.215422	0.222147	0.200769	0.150344
0.641966	0.901437	1.63829	0.982169	0.20744	1.19029	2.08859	0.860513	1.43205
0.440818	0.417623	0.192446	1.42156	0.276458	0.208623	0.64538	0.590463	0.047429
0.076813	0.017374	0.022706	0.377191	0.027573	0.093423	0.024071	0	0.03726
0	0.158045	0.03464	0	0	0	0	0.231364	0.098832
0.174186	0.15101	0.265519	0	0.085695	0.045048	0.400206	0.489526	0.14549
0	0.304164	0.241396	0.134603	0.020071	0.12033	0.502527	0.311805	0.161038
0.362907	0.358915	0	1.47065	0	0	0.367376	0.747718	0
26.9831	17.1892	27.4511	13.4576	8.25825	28.1172	12.5158	23.9942	35.8213
14.3542	13.0642	10.6662	11.2311	9.80893	25.6733	20.7584	20.0991	17.9741
0.244065	0.071268	0.133609	0.106844	0.168634	0	0	0	0.023386
0.117595	0	0.117504	2.15331	0	0	0.064238	0.061347	0
1.27028	0.380052	2.47685	0.292167	0.43389	0.780832	0.338872	1.19456	0.085145
27.207	10.1206	14.6913	11.3085	6.97508	7.366	11.801	17.1693	8.0394
0.031256	0.111556	0.096993	0	0.073942	0.040803	0.133821	0.321262	0.0303
0.003189	0.07503	0.234179	0.092967	0.097156	0.03705	0.131883	0.083507	0.354775
0.618511	0.773972	1.21469	0.275948	0.390252	0.32146	3.05324	1.1857	0.100913
1.40226	0.950792	1.08398	0.225271	0.851884	0.298728	0.717585	1.41654	0.145447
0.105234	0.077656	0.536522	0	0.015811	0.018282	0.078396	0.182009	0
0	1.32849	1.95219	0.146083	0.017367	0.013218	1.50087	0.649817	0.125725
0.702353	4.02107	1.2288	1.05433	0.260047	1.15283	1.26549	1.24695	3.92166
0.169649	0.478955	0.43766	0.198506	0.13107	0.039715	0.793904	0.401594	0.137794
0.033781	0.372977	0.058163	0.038629	0.309888	0.087618	0.191626	0.308972	0.020948
0.082616	0.287209	0.17248	0.246173	0.335147	0.492813	0.299162	0.221964	0.16501
162.305	153.854	207.451	187.344	49.9341	318.201	239.573	454.593	218.337
1.51099	1.4508	2.18282	0.302115	0.149985	0.477622	1.25589	0.991478	1.12153
0.139939	0.625295	0.413761	0.114129	0.241003	0.181039	0.699311	0.842069	0.111639

TARGET-4	TARGET-4	TARGET-4	TARGET-4	TARGET-4	TARGET-4	TARGET-4	TARGET-4	TARGET-4
0.071583	0.68559	0.047235	0.527726	0.582725	0	0	0.367704	0.126199
0.191299	0.10056	0.298062	0.476437	1.83485	0.379108	0	1.7935	0.305228
0	0.246184	0.304042	0	0	0.0631	0	0.355136	0.082084
2.60426	1.70931	3.73244	1.56674	0	4.95637	2.50633	1.96313	5.20758
12.1313	3.14596	4.75062	22.5797	15.1551	5.99371	4.56586	6.6017	5.29045
0.313507	0.514741	0.346007	0.291446	0.146187	0.230247	0.040791	0.240555	0.100828
1.37646	4.49566	1.39829	2.26337	1.09705	0.766353	2.0171	1.56183	1.526
0	0.265845	0.465904	0.803503	0.047562	0.442347	0.10552	0.120205	0.086341
0.754824	2.3298	2.08674	2.64324	0.543352	1.24039	1.43922	1.103	1.21888
1.08123	2.95131	0.535356	0.843769	0.220926	0.562435	0.472497	0.620407	0.891106
0.5018	0.058417	1.15276	0.057685	0.095912	0.041836	0	0.042928	0.330145
0	0	0.102397	0	0	0	0	0	0.222365
0.088674	0.03615	0.090145	0.086326	0.313314	0.306192	0.503852	0.100153	0.09181
0.344235	0.109489	0.235502	0.303761	0.709033	0.086984	0.152937	0.132332	0.173601
0.249362	2.01563	0	1.76839	0.666882	0.415162	0	0	0.10326
20.8185	25.2246	51.7608	79.7861	79.5485	18.0161	78.1544	44.528	37.8079
12.3849	13.2228	14.612	17.7791	20.8483	14.6728	15.9947	14.5827	25.5944
0.473098	0	0.085967	0	0.056392	0.160129	0	0.014746	0.503205
0.416567	0.085006	0	0	0	0.109298	0	0	0.101319
0.208844	0.671274	1.32863	0.119021	4.29097	0.834454	0.303033	0.217378	0.731706
14.1471	21.0763	6.02525	4.9713	9.89459	16.011	15.4004	1.55422	14.1873
0.185051	0	0.070844	0.007403	0.169392	0.011219	0.02515	0.008818	0.011223
0.00856	0.091925	0.091669	0.090241	0.063059	0.021299	0.064455	0.057602	0.006024
0.95051	0.489151	0.670268	1.58209	16.5666	1.32698	1.77308	2.32396	1.83849
0.148721	4.53508	2.2598	0	0.502644	0.132135	0.702811	0.214475	1.5771
0.223367	0	0.973745	0.070775	0.049827	0.0048	0	0.071298	0.073761
0.435297	1.64261	0.026239	0.141511	0.257907	0.039169	0.591491	0.239925	0.563497
2.20154	3.21235	2.59301	3.00678	0.640332	2.26637	1.57833	1.22261	2.1186
0.19989	0	0.728538	0.648653	0.05626	0.042402	0.083475	0.236674	6.07026
0.131995	0.071701	0.206253	0.186743	0.749563	0.116984	0.116606	0.051169	0.13595
0.467443	1.24784	0.382917	0.480275	0.402691	0.265627	0.230447	0.442321	0.341105
253.833	414.783	146.35	282.883	156.099	343.811	174.189	151.254	179
1.31925	0.398115	3.00183	1.62017	0.702947	2.02923	1.85234	0.924602	1.00959
0.391016	0.621517	0.101467	0.396276	0.403397	0.145629	0.260272	0.245079	0.197754

TARGET-4	TARGET-4	TARGET-4	TARGET-4	TARGET-4	TARGET-4	TARGET-4	TARGET-4	TARGET-4
0.418631	1.82313	1.60435	0.179248	4.21728	0.26505	1.22238	0.012438	0.523345
0	0.459316	0.607049	0	0.520566	0.109362	0.152447	0.230694	0.100454
0.162753	0.595647	0	0.212618	0	0	0	0.107057	0
1.82573	4.13856	1.16007	0	0	0.372832	4.83056	0.086221	0.079349
2.76189	16.6582	7.88562	13.1184	5.50784	11.6941	5.90717	1.11694	4.32114
0.100515	0.074528	0.243022	0.371423	0.043504	0.069114	0.02305	0.04837	0.117191
18.9032	0.884362	3.31558	0.894197	2.01909	4.90697	1.59221	0.244687	0.740484
0.440904	0.074878	0.322751	0.146114	0.076621	0.474032	0.145087	0.227451	0.151731
2.57791	0.705864	1.67148	0.845476	1.20815	3.94013	0.849694	0.029801	0.786499
0.476402	0.59976	0.507994	0.220823	0.470595	0.39807	0.306249	0.018653	0.029023
0.684589	0	0.305484	0.028867	0.040251	0.312413	0	0.014073	0
1.78187	0	0	0.449086	0.115911	0	0	0.274538	0
0.633638	0.080246	0.458886	0.030915	1.66122	0.061465	0.350309	0.029267	0.290112
1.40436	0.190569	0.091686	0.32582	0.251341	0.749103	0.191014	0.187861	0
1.98263	0	0	0	0	0	0.232037	0	0
14.7257	49.4136	36.1526	21.0588	24.8653	7.17619	7.17074	14.1691	13.7447
24.534	21.3521	28.6247	29.3952	40.1236	10.5263	7.91074	23.1533	113.933
0.624941	0	0.228861	0	0	0	0	0.012417	0
0.15877	0.038431	0	0	0	0	0	0.102485	0
1.39129	0.630196	0.179334	0.232351	0.331473	0.031602	0.708539	0.069957	0.036804
23.998	5.07368	8.00758	7.70375	18.2344	20.0727	3.66151	1.6385	6.80495
4.48396	0.64938	0.025669	0	0.115237	0	0.15238	0.006336	0.176611
0.78439	0.927725	0.069167	0	0.13654	0.00105	0.099176	0.411221	0.141749
1.08037	0.290175	1.1184	0.482921	5.85245	7.06944	0.920708	0.065201	0.075673
2.05452	0.450631	0.653754	0.115133	0.815937	0.142389	0.441823	0.089968	0.369259
2.24866	0.036733	0	0	0	0	0	0	0.004918
0.291193	0.132125	0.4801	0	1.6061	1.17628	0.245793	0.030078	0.361607
2.23081	2.54753	3.99698	0.838385	1.88754	0.582126	2.49479	0.030399	0.393946
2.24323	0.127396	0.509885	0.062202	0	6.36449	0.107511	0.030742	0.270198
1.06776	0.009736	0.055079	0.048139	0.580809	0.103785	0.016458	0.014938	0.107584
0.874453	0.081323	0.468961	0.079453	0.427978	0.462983	0.205257	0	0.454652
154.126	88.7409	153.78	304.15	98.9144	45.5704	138.673	193.354	501.208
2.65287	0.660006	1.2012	0.305845	2.04727	0.467686	0.515361	0.106734	0.291906
1.42493	0.174455	0.160324	0.283776	0.342012	3.07031	0.391321	0.189577	0.514309

TARGET-4(	TARGET-4(	TARGET-40-PAVECB-01A-01D
0	0	0
0.517464	0.196645	1.00653
0.123275	0	0.202086
0	0.201594	1.29995
7.69418	4.81345	7.02613
0.125903	0	0
1.06475	0.379639	2.08272
0.067469	0.236158	0.127728
0.56683	5.34407	1.42715
0.782088	0.305818	0.958019
0.017949	0.021987	0.174059
0	0	0
0.106606	0.162605	0.082392
0.087105	0.105351	0.10267
0	0	0.805743
14.933	20.8378	17.6617
20.3926	10.8885	9.05671
0.26752	0	0.883063
1.41065	0.794767	0
0.299755	0	0.368403
5.40793	4.42539	7.56738
0.121022	0.078709	0.113055
0.003909	0.003335	0.038681
0.723832	0.592255	0.741641
0.066937	0	0.044869
0.201485	0	0
0.162179	1.38063	0.027404
1.9455	2.26118	0.407498
0.077697	0.052854	0.229758
0.085585	0.07303	0.126413
0.460306	0.04819	0.337536
163.188	135.532	147.913
0.314255	0.041604	1.1966
0.35339	0.118732	0.448454
