# Supplementary material for: Selection of lncRNAs That Influence the Prognosis of Osteosarcoma Based on Copy Number Variation Data
Source: J Oncol. 2022 Mar 26;2022:8024979. doi: 10.1155/2022/8024979 (PMC8976607; doi:10.1155/2022/8024979)
Supplement: Supplementary Materials — Supplementary Figure 1: GO function annotation and KEGG pathway enrichment analyses. (A) The bubble plots for GO function enrichment (biological process). The color of the dot stands for the different P values, and the size of the dot reflects the number of target genes enriched in the corresponding pathway. (B) The bar diagrams for KEGG pathways. The y-axis represents the pathways, and the x-axis represents enriched gene numbers, and the color means adjusted P value. Supplementary Table 1: lncRNAs with >30% CNV alteration rate. Supplementary Table 2: expression profiles of 34 CNV-lncRNAs in TCGA database. Supplementary Table 3: cis-regulatory relationships of 23 mRNAs and 16 CNV-lncRNAs. Supplementary Table 4: results of Pearson analysis of coding genes significantly associated with CNV-lncRNAs. Supplementary Table 5: results of GO and KEGG enrichment analysis of 294 coding genes significantly associated with CNV-lncRNAs. Supplementary Table 6: clinical information of high- and low-risk groups in the training set. Supplementary Table 7: clinical information for the high- and low-risk groups in the test set. Supplementary Table 8: GO enrichment analysis of risk score-related genes. Supplementary Table 9: KEGG enrichment analysis of risk score-related genes. [file 8024979.f1.zip › 8024979.f4.pdf]

chr	LncRNA id	start_Lnc	end_Lnc	NearGene id	start_NearGene	end_NearGene	distance
chr13	DLEU1	50082169	50528643	RPL34P26	50361378	50361811	-279209
chr08	DLGAP2	737628	1708476	LOC112268020	883265	897534	-145637
chr08	DLGAP2	737628	1708476	LOC105377777	879171	883226	-141543
chr13	DLEU1	50082169	50528643	ST13P4	50172018	50173616	-89849
chr24	PRORY	21381905	21451846	RBMY2TP	21430324	21437347	-48419
chr05	C5orf67	56511282	56606256	LOC105378978	56536830	56555886	-25548
chr13	DLEU1	50082169	50528643	RPL18P10	50099334	50099875	-17165
chr16	CAPN15	527712	554636	MIR3176	543277	543366	-15565
chr24	PRORY	21381905	21451846	RBMY2EP	21395148	21401562	-13243
chr22	ELFN2	37340644	37427479	LOC100506271	37352190	37354839	-11546
chr16	CAPN15	527712	554636	MIR5587	535316	535368	-7604
chr13	LMO7DN	75871038	75883813	LMO7DN-IT1	75876886	75881127	-5848
chr22	PRR34	46048531	46054225	PRR34-AS1	46053846	46058522	-5315
chr03	ARIH2OS	48917788	48919385	ARIH2	48918821	48986382	-1033
chr08	DLGAP2	737628	1708476	LOC401442	738548	740374	-920
chr10	C10orf55	73909969	73922777	PLAU	73909182	73917497	787
chr02	DIRC3	217284019	217756593	DIRC3-AS1	217282733	217344204	1286
chr06	C6orf223	44000585	44007612	SCIRT	43995723	44074652	4862
chr22	RFPL3S	32359906	32371264	RFPL3	32354885	32361161	5021
chr16	MEIOB	1833986	1872164	FAHD1	1827224	1840207	6762
chr02	DIRC1	188733738	188790123	LOC105373790	188655188	188751301	78550
chr02	CCDC140	222298147	222305217	PAX3	222199887	222298998	98260
chr13	DLEU1	50082169	50528643	DLEU2	49982549	50125541	99620
chr13	DLEU1	50082169	50528643	RPL34P26	50361378	50361811	-279209
