# Supplementary material for: Selection of lncRNAs That Influence the Prognosis of Osteosarcoma Based on Copy Number Variation Data
Source: J Oncol. 2022 Mar 26;2022:8024979. doi: 10.1155/2022/8024979 (PMC8976607; doi:10.1155/2022/8024979)
Supplement: Supplementary Materials — Supplementary Figure 1: GO function annotation and KEGG pathway enrichment analyses. (A) The bubble plots for GO function enrichment (biological process). The color of the dot stands for the different P values, and the size of the dot reflects the number of target genes enriched in the corresponding pathway. (B) The bar diagrams for KEGG pathways. The y-axis represents the pathways, and the x-axis represents enriched gene numbers, and the color means adjusted P value. Supplementary Table 1: lncRNAs with >30% CNV alteration rate. Supplementary Table 2: expression profiles of 34 CNV-lncRNAs in TCGA database. Supplementary Table 3: cis-regulatory relationships of 23 mRNAs and 16 CNV-lncRNAs. Supplementary Table 4: results of Pearson analysis of coding genes significantly associated with CNV-lncRNAs. Supplementary Table 5: results of GO and KEGG enrichment analysis of 294 coding genes significantly associated with CNV-lncRNAs. Supplementary Table 6: clinical information of high- and low-risk groups in the training set. Supplementary Table 7: clinical information for the high- and low-risk groups in the test set. Supplementary Table 8: GO enrichment analysis of risk score-related genes. Supplementary Table 9: KEGG enrichment analysis of risk score-related genes. [file 8024979.f1.zip › 8024979.f5.pdf]

| gene | coding genes | cor | p.value |
|---|---|---|---|
| AL023806.1 | ZNF670-ZNF695 | 0.865695981 | 1.63E-31 |
| AL023806.1 | UNC80 | 0.845894001 | 8.88E-29 |
| TMEM78 | OR4D9 | 0.844298195 | 1.42E-28 |
| AL023806.1 | LHFPL5 | 0.844228305 | 1.45E-28 |
| AL023806.1 | OR5AS1 | 0.839720443 | 5.29E-28 |
| AL023806.1 | C17orf105 | 0.835200932 | 1.87E-27 |
| TMEM78 | OR7G2 | 0.834641817 | 2.18E-27 |
| AL023806.1 | TMEM212 | 0.83049505 | 6.66E-27 |
| AL023806.1 | NPAP1 | 0.82905067 | 9.76E-27 |
| AL023806.1 | OR1A1 | 0.826651136 | 1.83E-26 |
| C3orf36 | SLCO2A1 | 0.82474364 | 2.99E-26 |
| TMEM78 | NANOG | 0.824484566 | 3.20E-26 |
| TMEM78 | KLB | 0.822457775 | 5.36E-26 |
| CCDC140 | PAX3 | 0.822297353 | 5.58E-26 |
| AL023806.1 | ERVW-1 | 0.821712289 | 6.46E-26 |
| AL023806.1 | CFAP54 | 0.821560862 | 6.72E-26 |
| AL023806.1 | NPIPB4 | 0.820803727 | 8.12E-26 |
| AL023806.1 | B4GALNT2 | 0.817320909 | 1.92E-25 |
| TMEM78 | OPRM1 | 0.814478742 | 3.83E-25 |
| TMEM78 | OR10G3 | 0.813919627 | 4.39E-25 |
| AL023806.1 | TPRXL | 0.813675015 | 4.65E-25 |
| TMEM78 | OR1I1 | 0.813651718 | 4.68E-25 |
| AL023806.1 | PLEKHD1 | 0.811298777 | 8.18E-25 |
| AL023806.1 | OR14J1 | 0.807582994 | 1.95E-24 |
| TMEM78 | TMEM213 | 0.807186954 | 2.14E-24 |
| TMEM78 | OR52I1 | 0.806802563 | 2.33E-24 |
| AL023806.1 | OR2T12 | 0.805742574 | 2.98E-24 |
| AL023806.1 | PDE6A | 0.805579499 | 3.09E-24 |
| AL023806.1 | OPHN1 | 0.804414677 | 4.03E-24 |
| TMEM78 | CRX | 0.800361095 | 1.00E-23 |
| AL023806.1 | LCN15 | 0.798311008 | 1.57E-23 |
| C8orf86 | ONECUT3 | 0.798217719 | 1.60E-23 |
| AL023806.1 | OR5A2 | 0.798124636 | 1.64E-23 |
| TMEM78 | OR7D4 | 0.797996506 | 1.68E-23 |
| TMEM78 | OR10H1 | 0.797518928 | 1.87E-23 |
| TMEM78 | OR2V2 | 0.796397194 | 2.38E-23 |
| TMEM78 | OR1M1 | 0.79501456 | 3.21E-23 |
| FAM106A | CCDC144A | 0.792242283 | 5.81E-23 |
| AL023806.1 | CDKL5 | 0.791287129 | 7.10E-23 |
| TMEM78 | SAG | 0.790005824 | 9.30E-23 |
| AL023806.1 | OR4F17 | 0.789784508 | 9.74E-23 |
| TMEM78 | OR1A1 | 0.789353524 | 1.07E-22 |
| AL023806.1 | OR6B1 | 0.789132207 | 1.12E-22 |
| TMEM78 | MTRNR2L4 | 0.788316832 | 1.32E-22 |
| AL023806.1 | ZNF483 | 0.788048923 | 1.40E-22 |
| AL023806.1 | FCRL5 | 0.787827606 | 1.46E-22 |
| AL023806.1 | C2orf81 | 0.787757717 | 1.48E-22 |
| AL023806.1 | SLC22A25 | 0.787198602 | 1.67E-22 |
| AL023806.1 | RGPD5 | 0.785894001 | 2.18E-22 |
| AL023806.1 | ZNF674 | 0.785567851 | 2.33E-22 |

| | | | |
|---|---|---|---|
| TMEM78 | EREG | 0.78543972 | 2.39E-22 |
| C8orf86 | OR4D1 | 0.785066109 | 2.58E-22 |
| AL023806.1 | OR6Y1 | 0.784740827 | 2.76E-22 |
| TMEM78 | NIM1K | 0.783273151 | 3.71E-22 |
| AL023806.1 | SZT2 | 0.782981945 | 3.93E-22 |
| AL023806.1 | NANOG | 0.782632499 | 4.22E-22 |
| CABIN1 | TOP3B | 0.78157251 | 5.22E-22 |
| C8orf86 | C1orf64 | 0.781385056 | 5.42E-22 |
| AL023806.1 | SMG1 | 0.781234712 | 5.59E-22 |
| TMEM78 | SLC1A6 | 0.781141526 | 5.69E-22 |
| TMEM78 | FRMPD1 | 0.780885265 | 5.99E-22 |
| TMEM78 | POU5F2 | 0.780465929 | 6.51E-22 |
| TMEM78 | OR14J1 | 0.779720443 | 7.55E-22 |
| AL023806.1 | EFCAB3 | 0.779685498 | 7.60E-22 |
| C8orf86 | OR1M1 | 0.779439689 | 7.98E-22 |
| AL023806.1 | RGR | 0.778870122 | 8.93E-22 |
| AL023806.1 | OR2V2 | 0.778651074 | 9.33E-22 |
| TMEM78 | TMEM40 | 0.777192778 | 1.24E-21 |
| TMEM78 | FADS6 | 0.776622015 | 1.39E-21 |
| TMEM78 | OPRD1 | 0.776610367 | 1.39E-21 |
| TMEM78 | UGT1A1 | 0.775829936 | 1.62E-21 |
| CABIN1 | PISD | 0.775678509 | 1.67E-21 |
| AL023806.1 | TTC34 | 0.775340711 | 1.78E-21 |
| TMEM78 | CYP1A2 | 0.77396622 | 2.32E-21 |
| AL023806.1 | HTN3 | 0.773791497 | 2.40E-21 |
| AL023806.1 | MYLK3 | 0.773162493 | 2.71E-21 |
| AL023806.1 | SHC4 | 0.772987769 | 2.80E-21 |
| AL023806.1 | FAM129C | 0.772824694 | 2.89E-21 |
| TMEM78 | MOGAT3 | 0.772754805 | 2.93E-21 |
| AL023806.1 | KLB | 0.77271986 | 2.95E-21 |
| AL023806.1 | IGFL4 | 0.771788002 | 3.52E-21 |
| AL023806.1 | KIAA1328 | 0.771624927 | 3.63E-21 |
| TMEM78 | OR7A17 | 0.768910891 | 6.06E-21 |
| TMEM78 | HRH4 | 0.768899243 | 6.08E-21 |
| AL023806.1 | NKTR | 0.768177053 | 6.96E-21 |
| TMEM78 | IGFL4 | 0.767419918 | 8.01E-21 |
| AL023806.1 | ATP10B | 0.76616191 | 1.01E-20 |
| TMEM78 | CDKL5 | 0.765870705 | 1.07E-20 |
| AL023806.1 | C1orf64 | 0.765649388 | 1.11E-20 |
| TMEM78 | C2orf81 | 0.76498544 | 1.26E-20 |
| C8orf86 | OR4D9 | 0.764948455 | 1.27E-20 |
| AL023806.1 | NXNL2 | 0.763645894 | 1.61E-20 |
| TMEM78 | OR5AS1 | 0.76341293 | 1.68E-20 |
| AL023806.1 | PATE4 | 0.763401281 | 1.68E-20 |
| AL023806.1 | OR10H1 | 0.762970297 | 1.82E-20 |
| AL023806.1 | OR1C1 | 0.762730423 | 1.90E-20 |
| C8orf86 | AL357140.1 | 0.762662505 | 1.92E-20 |
| C8orf86 | CYP1A2 | 0.762443941 | 2.00E-20 |
| TMEM78 | CFAP54 | 0.762376238 | 2.02E-20 |
| AL023806.1 | PROM2 | 0.761118229 | 2.54E-20 |
| C8orf86 | NANOG | 0.760766499 | 2.71E-20 |

| | | | |
|---|---|---|---|
| AL023806.1 | LA16c-431H6.6 | 0.760710542 | 2.74E-20 |
| TMEM78 | UQCRHL | 0.760384391 | 2.90E-20 |
| C8orf86 | MYLK3 | 0.759997672 | 3.11E-20 |
| AL023806.1 | OR10G3 | 0.759138031 | 3.63E-20 |
| TMEM78 | ONECUT3 | 0.758974956 | 3.73E-20 |
| TMEM78 | OR2T12 | 0.758846826 | 3.82E-20 |
| PSMB1 | PDCD2 | 0.757775189 | 4.62E-20 |
| TMEM78 | UNC80 | 0.7577053 | 4.68E-20 |
| TMEM78 | SGK3 | 0.757612114 | 4.76E-20 |
| AL023806.1 | MROH7 | 0.757588818 | 4.78E-20 |
| TMEM78 | OR4K13 | 0.757262667 | 5.06E-20 |
| AL023806.1 | OR2G6 | 0.757204426 | 5.11E-20 |
| CABIN1 | MED15 | 0.756994758 | 5.31E-20 |
| AL023806.1 | TMEM78 | 0.75685498 | 5.44E-20 |
| TMEM78 | AL023806.1 | 0.75685498 | 5.44E-20 |
| AL023806.1 | SPP2 | 0.756805554 | 5.49E-20 |
| TMEM78 | ZNF383 | 0.756691904 | 5.60E-20 |
| TMEM78 | OR5AU1 | 0.756622015 | 5.67E-20 |
| C8orf86 | NLRP2 | 0.756235076 | 6.07E-20 |
| TMEM78 | OR6C75 | 0.755818288 | 6.53E-20 |
| C8orf86 | CYP4F8 | 0.755710876 | 6.66E-20 |
| TMEM78 | C17orf105 | 0.75521258 | 7.26E-20 |
| C8orf86 | CRX | 0.754942048 | 7.62E-20 |
| AL023806.1 | TRAPPC3L | 0.754653465 | 8.01E-20 |
| AL023806.1 | FUT1 | 0.754513687 | 8.21E-20 |
| TMEM78 | ZNF674 | 0.754187536 | 8.69E-20 |
| AL023806.1 | ACSM2A | 0.75383809 | 9.24E-20 |
| TMEM78 | PLA2G4D | 0.753616773 | 9.60E-20 |
| AL023806.1 | NIM1K | 0.75344205 | 9.89E-20 |
| AL023806.1 | OR2M3 | 0.753418754 | 9.93E-20 |
| AL023806.1 | UGT1A1 | 0.75331392 | 1.01E-19 |
| AL023806.1 | GLB1L3 | 0.753092603 | 1.05E-19 |
| C8orf86 | OR14J1 | 0.752996681 | 1.07E-19 |
| TMEM78 | RGR | 0.752952825 | 1.08E-19 |
| TMEM78 | ATP5L2 | 0.752358765 | 1.19E-19 |
| AL023806.1 | OR4D9 | 0.752218987 | 1.22E-19 |
| TMEM78 | PNLIPRP1 | 0.75214621 | 1.24E-19 |
| CABIN1 | TRIOBP | 0.751834595 | 1.31E-19 |
| AL023806.1 | GOLGA8R | 0.751531741 | 1.38E-19 |
| AL023806.1 | KIR3DX1 | 0.751205591 | 1.46E-19 |
| CABIN1 | PPIL2 | 0.750564939 | 1.63E-19 |
| AL023806.1 | FER1L5 | 0.750529994 | 1.64E-19 |
| AL023806.1 | FADS6 | 0.750413512 | 1.67E-19 |
| TMEM78 | KCNA7 | 0.750273733 | 1.71E-19 |
| TMEM78 | ZNF292 | 0.750157251 | 1.74E-19 |
| TMEM78 | RP11-310N16.1 | 0.75009537 | 1.76E-19 |
| TMEM78 | CCL16 | 0.749761211 | 1.87E-19 |
| AL023806.1 | FER | 0.74950495 | 1.95E-19 |
| AL023806.1 | FSIP2 | 0.749388468 | 1.99E-19 |
| AL023806.1 | CHP2 | 0.748596389 | 2.28E-19 |
| TMEM78 | SLC19A3 | 0.748596389 | 2.28E-19 |

| | | | |
|---|---|---|---|
| TMEM78 | OR5H14 | 0.748584741 | 2.28E-19 |
| AL023806.1 | NEDD4 | 0.748468259 | 2.33E-19 |
| AL023806.1 | CYP3A5 | 0.748142108 | 2.46E-19 |
| TMEM78 | OR2AT4 | 0.748118812 | 2.47E-19 |
| TMEM78 | GLB1L3 | 0.748072219 | 2.49E-19 |
| AL023806.1 | SLC1A6 | 0.747885847 | 2.57E-19 |
| AL023806.1 | OR2M4 | 0.747687828 | 2.65E-19 |
| TMEM78 | SH2D3A | 0.747513104 | 2.73E-19 |
| AL023806.1 | CA6 | 0.747093768 | 2.93E-19 |
| AL023806.1 | OR1M1 | 0.746476412 | 3.26E-19 |
| AL023806.1 | IQCF3 | 0.74616191 | 3.43E-19 |
| AL023806.1 | MTRNR2L4 | 0.745626092 | 3.76E-19 |
| AL023806.1 | SGK3 | 0.745497962 | 3.84E-19 |
| TMEM78 | CCDC79 | 0.744834013 | 4.29E-19 |
| AL023806.1 | KCNH1 | 0.744158416 | 4.80E-19 |
| AL023806.1 | POU5F2 | 0.744100175 | 4.84E-19 |
| AL023806.1 | ZNF292 | 0.743948748 | 4.97E-19 |
| AL023806.1 | OR7D4 | 0.743808969 | 5.08E-19 |
| TMEM78 | AL357140.1 | 0.743677853 | 5.20E-19 |
| C8orf86 | OR1A1 | 0.743654262 | 5.22E-19 |
| AL023806.1 | TMEM266 | 0.74262085 | 6.19E-19 |
| AL023806.1 | OR1D2 | 0.741793826 | 7.10E-19 |
| TMEM78 | PLEKHD1 | 0.741595807 | 7.33E-19 |
| TMEM78 | TMEM212 | 0.741059988 | 8.00E-19 |
| AL023806.1 | OR5A1 | 0.741036692 | 8.04E-19 |
| TMEM78 | MYLK3 | 0.741036692 | 8.04E-19 |
| AL023806.1 | GPR83 | 0.74039604 | 8.92E-19 |
| AL023806.1 | ZNF789 | 0.740337798 | 9.01E-19 |
| C8orf86 | PCDHA9 | 0.740217836 | 9.19E-19 |
| AL023806.1 | TMEM213 | 0.739965055 | 9.58E-19 |
| AL023806.1 | CCL16 | 0.739615609 | 1.01E-18 |
| AL023806.1 | GPX2 | 0.739542049 | 1.03E-18 |
| C8orf86 | OR2V2 | 0.739398741 | 1.05E-18 |
| TMEM78 | NPAP1 | 0.739138031 | 1.10E-18 |
| TMEM78 | NCMAP | 0.738578917 | 1.20E-18 |
| TMEM78 | SLC22A25 | 0.738345952 | 1.25E-18 |
| TMEM78 | NKTR | 0.737880023 | 1.34E-18 |
| C8orf86 | SLC26A8 | 0.737771566 | 1.37E-18 |
| AL023806.1 | EBLN2 | 0.737577169 | 1.41E-18 |
| TMEM78 | KCNH1 | 0.737518928 | 1.42E-18 |
| C8orf86 | NCMAP | 0.737457046 | 1.44E-18 |
| AL023806.1 | TMC2 | 0.737425743 | 1.45E-18 |
| AL023806.1 | SLFN12L | 0.737390798 | 1.45E-18 |
| AL023806.1 | ZNF611 | 0.736924869 | 1.57E-18 |
| C8orf86 | TMEM239 | 0.736385347 | 1.71E-18 |
| TMEM78 | ATP10B | 0.7360629 | 1.80E-18 |
| AL023806.1 | ZNF564 | 0.736027956 | 1.81E-18 |
| TMEM78 | CDC20B | 0.736027956 | 1.81E-18 |
| TMEM78 | CFTR | 0.736004659 | 1.82E-18 |
| AL023806.1 | ZDHHC15 | 0.735876529 | 1.85E-18 |
| C8orf86 | C2orf81 | 0.73572136 | 1.90E-18 |

| | | | |
|---|---|---|---|
| C8orf86 | SAG | 0.735674764 | 1.92E-18 |
| C8orf86 | MOGAT3 | 0.735430137 | 1.99E-18 |
| TMEM78 | CLEC19A | 0.735072801 | 2.11E-18 |
| C8orf86 | RD3 | 0.734717652 | 2.23E-18 |
| AL023806.1 | OR2AG1 | 0.734513687 | 2.31E-18 |
| C8orf86 | ZIM3 | 0.734413588 | 2.34E-18 |
| TMEM78 | FBXW12 | 0.734269074 | 2.40E-18 |
| TMEM78 | LRRC74B | 0.734234129 | 2.41E-18 |
| C8orf86 | VWA5B1 | 0.73402062 | 2.49E-18 |
| AL023806.1 | OR1I1 | 0.733954572 | 2.52E-18 |
| TMEM78 | KCNC1 | 0.733950885 | 2.52E-18 |
| TMEM78 | CYP4F8 | 0.733942924 | 2.52E-18 |
| C3orf36 | RAMP3 | 0.733875111 | 2.55E-18 |
| AL023806.1 | NPIPB5 | 0.73364007 | 2.65E-18 |
| C8orf86 | TMEM213 | 0.733414877 | 2.75E-18 |
| AL023806.1 | MOGAT3 | 0.733197437 | 2.84E-18 |
| CABIN1 | LZTR1 | 0.733080955 | 2.89E-18 |
| TMEM78 | OR2G6 | 0.733069307 | 2.90E-18 |
| FAM106A | B4GALNT2 | 0.732894584 | 2.98E-18 |
| TMEM78 | TMC2 | 0.732894584 | 2.98E-18 |
| AL023806.1 | CCDC122 | 0.732882935 | 2.99E-18 |
| AL023806.1 | OR10G4 | 0.732813046 | 3.02E-18 |
| CABIN1 | PI4KA | 0.732754805 | 3.05E-18 |
| AL023806.1 | HSD17B13 | 0.732626674 | 3.11E-18 |
| AL023806.1 | FBXO24 | 0.73252184 | 3.16E-18 |
| TMEM78 | OR5A1 | 0.732463599 | 3.19E-18 |
| AL023806.1 | GSG1 | 0.732451951 | 3.20E-18 |
| AL023806.1 | OR10K1 | 0.732440303 | 3.20E-18 |
| C8orf86 | FAM129C | 0.73242472 | 3.21E-18 |
| AL023806.1 | OR5AU1 | 0.731776354 | 3.56E-18 |
| TMEM78 | AC087762.1 | 0.731403611 | 3.77E-18 |
| AL023806.1 | GOLGA6L4 | 0.730728014 | 4.19E-18 |
| AL023806.1 | AC087762.1 | 0.730704718 | 4.21E-18 |
| C8orf86 | OR6Y1 | 0.730491002 | 4.35E-18 |
| AL023806.1 | ZNF225 | 0.730308678 | 4.48E-18 |
| TMEM78 | SMG1 | 0.730040769 | 4.67E-18 |
| TMEM78 | MEFV | 0.729866045 | 4.80E-18 |
| AL023806.1 | REV3L | 0.729749563 | 4.88E-18 |
| C8orf86 | BEND2 | 0.729500846 | 5.08E-18 |
| TMEM78 | GP2 | 0.729315426 | 5.23E-18 |
| AL023806.1 | ZNF383 | 0.729260338 | 5.27E-18 |
| AL023806.1 | OR2L2 | 0.7291788 | 5.34E-18 |
| AL023806.1 | KCNA7 | 0.729015725 | 5.47E-18 |
| AL023806.1 | ZNF141 | 0.72885265 | 5.62E-18 |
| TMEM78 | OR51M1 | 0.727936747 | 6.47E-18 |
| AL023806.1 | AP3B2 | 0.727641235 | 6.77E-18 |
| AL023806.1 | TEX35 | 0.727582994 | 6.83E-18 |
| TMEM78 | NCCRP1 | 0.727571345 | 6.85E-18 |
| C8orf86 | KCNH1 | 0.726611918 | 7.94E-18 |
| AL023806.1 | CFTR | 0.726534653 | 8.03E-18 |
| C8orf86 | OR1I1 | 0.726507078 | 8.07E-18 |

| | | | |
|---|---|---|---|
| C8orf86 | DAND5 | 0.726402238 | 8.20E-18 |
| TMEM78 | NUGGC | 0.726266744 | 8.37E-18 |
| CABIN1 | ZDHHC8 | 0.726173559 | 8.49E-18 |
| C8orf86 | TMEM105 | 0.726134313 | 8.54E-18 |
| AL023806.1 | OPRM1 | 0.725975539 | 8.75E-18 |
| C8orf86 | OR7G2 | 0.725971228 | 8.76E-18 |
| TMEM78 | MAS1 | 0.725824112 | 8.96E-18 |
| C8orf86 | OR5A1 | 0.725761548 | 9.04E-18 |
| TMEM78 | C1orf64 | 0.725719278 | 9.10E-18 |
| TMEM78 | LCN15 | 0.725695981 | 9.14E-18 |
| C8orf86 | TMEM212 | 0.725645059 | 9.21E-18 |
| AL023806.1 | C9orf84 | 0.725614444 | 9.25E-18 |
| AL023806.1 | ZNF440 | 0.725579499 | 9.30E-18 |
| TMEM78 | KCNG4 | 0.724799068 | 1.05E-17 |
| TMEM78 | OR10J1 | 0.724740827 | 1.06E-17 |
| AL023806.1 | SYCP1 | 0.724713817 | 1.06E-17 |
| C8orf86 | AP3B2 | 0.724538413 | 1.09E-17 |
| TMEM78 | ZNF891 | 0.724437973 | 1.11E-17 |
| AL023806.1 | TMEM182 | 0.724414677 | 1.11E-17 |
| AL023806.1 | DNAH17 | 0.724298195 | 1.13E-17 |
| AL023806.1 | CNTN2 | 0.723972044 | 1.19E-17 |
| AL023806.1 | OR4K13 | 0.723552708 | 1.27E-17 |
| AL023806.1 | STRC | 0.723075131 | 1.36E-17 |
| AL023806.1 | OR2T33 | 0.722679091 | 1.45E-17 |
| TMEM78 | LHFPL5 | 0.72262085 | 1.46E-17 |
| AL023806.1 | C2orf80 | 0.722582674 | 1.47E-17 |
| AL023806.1 | NUGGC | 0.722574257 | 1.47E-17 |
| TMEM78 | LIN28A | 0.722527665 | 1.48E-17 |
| AL023806.1 | STARD9 | 0.722387886 | 1.51E-17 |
| TMEM78 | SLC7A14 | 0.722317997 | 1.53E-17 |
| TMEM78 | KCTD19 | 0.722259755 | 1.54E-17 |
| AL023806.1 | ZNF169 | 0.722119977 | 1.57E-17 |
| CABIN1 | DGCR14 | 0.722119977 | 1.57E-17 |
| TMEM78 | OR2M3 | 0.722108328 | 1.58E-17 |
| TMEM78 | OR5A2 | 0.721386139 | 1.76E-17 |
| AL023806.1 | HTRA4 | 0.721153174 | 1.82E-17 |
| AL023806.1 | MYCBPAP | 0.721141526 | 1.82E-17 |
| AL023806.1 | SLC5A12 | 0.72104834 | 1.85E-17 |
| PSMB1 | MRPL18 | 0.721032967 | 1.85E-17 |
| TMEM78 | OR2AG1 | 0.720908561 | 1.89E-17 |
| AL023806.1 | ITIH1 | 0.720708233 | 1.94E-17 |
| TMEM78 | RICTOR | 0.720629004 | 1.97E-17 |
| TMEM78 | OR6Y1 | 0.720559115 | 1.99E-17 |
| TMEM78 | PRR23C | 0.720489225 | 2.01E-17 |
| C8orf86 | OR4K13 | 0.720216671 | 2.09E-17 |
| TMEM78 | PRDM14 | 0.72012813 | 2.12E-17 |
| CABIN1 | SMARCB1 | 0.720034945 | 2.15E-17 |
| AL023806.1 | DNASE1 | 0.719790332 | 2.23E-17 |
| AL023806.1 | IDO2 | 0.719767036 | 2.24E-17 |
| TMEM78 | ZSCAN4 | 0.719569016 | 2.31E-17 |
| AL023806.1 | OR5H14 | 0.719417589 | 2.36E-17 |

| | | | |
|---|---|---|---|
| TMEM78 | KIR3DX1 | 0.719405941 | 2.36E-17 |
| CABIN1 | DGCR2 | 0.719394292 | 2.37E-17 |
| C8orf86 | TAT | 0.719343003 | 2.38E-17 |
| AL023806.1 | ONECUT3 | 0.719196273 | 2.44E-17 |
| AL023806.1 | LRRC74B | 0.719138031 | 2.46E-17 |
| TMEM78 | NOTO | 0.71907979 | 2.48E-17 |
| TMEM78 | OR7A5 | 0.719009901 | 2.51E-17 |
| FAM106A | ZNF169 | 0.718974956 | 2.52E-17 |
| AL023806.1 | CRX | 0.71895166 | 2.53E-17 |
| TMEM78 | GPR83 | 0.718928363 | 2.54E-17 |
| TMEM78 | NSRP1 | 0.718858474 | 2.56E-17 |
| AL023806.1 | OR7A5 | 0.718730344 | 2.61E-17 |
| AL023806.1 | ZNF546 | 0.718590565 | 2.67E-17 |
| AL023806.1 | ATP5L2 | 0.718474083 | 2.71E-17 |
| TMEM78 | OR1D2 | 0.718380897 | 2.75E-17 |
| AL023806.1 | EFCAB12 | 0.718112988 | 2.86E-17 |
| AL023806.1 | RNF222 | 0.718008154 | 2.91E-17 |
| AL023806.1 | OR51M1 | 0.717941553 | 2.94E-17 |
| AL023806.1 | SPDYE5 | 0.717751893 | 3.02E-17 |
| AL023806.1 | OR2AT4 | 0.717414094 | 3.17E-17 |
| TMEM78 | OR10H5 | 0.717367501 | 3.20E-17 |
| TMEM78 | PADI4 | 0.717276404 | 3.24E-17 |
| C8orf86 | AIPL1 | 0.716968715 | 3.39E-17 |
| C8orf86 | OR6C75 | 0.716698702 | 3.53E-17 |
| C8orf86 | NUGGC | 0.716314289 | 3.73E-17 |
| TMEM78 | SULT2A1 | 0.715853232 | 3.99E-17 |
| TMEM78 | IL12B | 0.715794991 | 4.03E-17 |
| CAPN15 | AXIN1 | 0.715771695 | 4.04E-17 |
| TMEM78 | NLRP12 | 0.715119394 | 4.45E-17 |
| TMEM78 | IFNW1 | 0.715065127 | 4.48E-17 |
| TMEM78 | CEP152 | 0.715049505 | 4.49E-17 |
| TMEM78 | NPIPB4 | 0.714967967 | 4.55E-17 |
| AL023806.1 | PRDM7 | 0.71481654 | 4.65E-17 |
| AL023806.1 | EPPIN | 0.714793244 | 4.66E-17 |
| TMEM78 | OR8G1 | 0.714556955 | 4.83E-17 |
| C8orf86 | PLA2G4D | 0.71439222 | 4.94E-17 |
| AL023806.1 | HRH4 | 0.714071054 | 5.18E-17 |
| TMEM78 | GLRA1 | 0.714056075 | 5.19E-17 |
| C8orf86 | OR8G1 | 0.713829738 | 5.37E-17 |
| TMEM78 | ZNF483 | 0.713744904 | 5.43E-17 |
| TMEM78 | CA6 | 0.713733256 | 5.44E-17 |
| C8orf86 | RHBG | 0.713693286 | 5.47E-17 |
| C8orf86 | MTRNR2L4 | 0.713297223 | 5.80E-17 |
| C8orf86 | KIR3DX1 | 0.713285574 | 5.81E-17 |
| TMEM78 | MYL10 | 0.712786404 | 6.24E-17 |
| AL023806.1 | CARD14 | 0.712335469 | 6.66E-17 |
| AL023806.1 | ZNF891 | 0.712265579 | 6.73E-17 |
| C8orf86 | OR7A17 | 0.712062439 | 6.93E-17 |
| C8orf86 | CA6 | 0.711817812 | 7.18E-17 |
| TMEM78 | TMEM239 | 0.711636575 | 7.37E-17 |
| CABIN1 | KLHL22 | 0.711438556 | 7.59E-17 |

| | | | |
|---|---|---|---|
| TMEM78 | GCNT3 | 0.711380315 | 7.65E-17 |
| TMEM78 | MOBP | 0.711182295 | 7.87E-17 |
| AL023806.1 | SLC34A2 | 0.710984275 | 8.10E-17 |
| AL023806.1 | BLOC1S6 | 0.710891089 | 8.21E-17 |
| AL023806.1 | CRLF3 | 0.710879441 | 8.22E-17 |
| AL023806.1 | ZNF345 | 0.710832848 | 8.28E-17 |
| TMEM78 | VWA5B1 | 0.710588235 | 8.57E-17 |
| C8orf86 | OR2B11 | 0.710513135 | 8.66E-17 |
| TMEM78 | AP3B2 | 0.710366919 | 8.85E-17 |
| C8orf86 | OR5AS1 | 0.710338402 | 8.88E-17 |
| TMEM78 | FLG2 | 0.710215492 | 9.04E-17 |
| C8orf86 | ATP10B | 0.710047179 | 9.26E-17 |
| TMEM78 | DAND5 | 0.709982528 | 9.35E-17 |
| AL023806.1 | FLG2 | 0.709889342 | 9.47E-17 |
| TMEM78 | B4GALNT2 | 0.709819453 | 9.57E-17 |
| C8orf86 | UGT1A1 | 0.709779255 | 9.62E-17 |
| TMEM78 | CTCFL | 0.709644729 | 9.81E-17 |
| AL023806.1 | OR52K1 | 0.709551543 | 9.94E-17 |
| TMEM78 | AL356289.1 | 0.709501192 | 1.00E-16 |
| AL023806.1 | C6orf25 | 0.709365172 | 1.02E-16 |
| AL023806.1 | GABRP | 0.709085614 | 1.06E-16 |
| AL023806.1 | SLC35E2 | 0.708444962 | 1.16E-16 |
| TMEM78 | CACNA1A | 0.708351776 | 1.18E-16 |
| CABIN1 | TAOK2 | 0.708305183 | 1.19E-16 |
| TMEM78 | OR4F15 | 0.707754143 | 1.28E-16 |
| TMEM78 | CYP4F3 | 0.707291788 | 1.37E-16 |
| FAM106A | SMG1 | 0.707093768 | 1.41E-16 |
| AL023806.1 | RDH16 | 0.707058824 | 1.42E-16 |
| AL023806.1 | GLRA1 | 0.706869038 | 1.46E-16 |
| C8orf86 | OR5H14 | 0.706773838 | 1.47E-16 |
| C8orf86 | NRSN1 | 0.706238907 | 1.59E-16 |
| AL023806.1 | UBR1 | 0.70616191 | 1.61E-16 |
| AL023806.1 | BCL2L14 | 0.706103669 | 1.62E-16 |
| AL023806.1 | ARL17A | 0.706092021 | 1.62E-16 |
| C10orf55 | ARHGAP22 | 0.706010483 | 1.64E-16 |
| AL023806.1 | ESR2 | 0.705521258 | 1.76E-16 |
| AL023806.1 | ART3 | 0.705451369 | 1.78E-16 |
| AL023806.1 | FRMPD1 | 0.705358183 | 1.80E-16 |
| TMEM78 | MYH7B | 0.705241701 | 1.83E-16 |
| AL023806.1 | PROX2 | 0.704900131 | 1.92E-16 |
| AL023806.1 | GFI1B | 0.704834013 | 1.94E-16 |
| C8orf86 | OR10H1 | 0.704828471 | 1.94E-16 |
| C8orf86 | ASB18 | 0.704583844 | 2.01E-16 |
| TMEM78 | RIMS2 | 0.704554455 | 2.01E-16 |
| TMEM78 | CHST9 | 0.704333139 | 2.08E-16 |
| AL023806.1 | PCDHA9 | 0.704111823 | 2.14E-16 |
| TMEM78 | CTD-3105H18.18 | 0.703983692 | 2.18E-16 |
| TMEM78 | RAX2 | 0.703980897 | 2.18E-16 |
| AL023806.1 | FBXO15 | 0.703599301 | 2.30E-16 |
| TMEM78 | CATIP | 0.703564356 | 2.31E-16 |
| TMEM78 | A2ML1 | 0.703552708 | 2.32E-16 |

| | | | |
|---|---|---|---|
| TMEM78 | MEIOC | 0.703296447 | 2.40E-16 |
| AL023806.1 | LRRD1 | 0.702981945 | 2.51E-16 |
| TMEM78 | ACP7 | 0.702923704 | 2.53E-16 |
| TMEM78 | LA16c-431H6.6 | 0.702585906 | 2.65E-16 |
| TMEM78 | BEND2 | 0.702481072 | 2.69E-16 |
| CAPN15 | RHOT2 | 0.702399534 | 2.72E-16 |
| AL023806.1 | FAM186A | 0.702131625 | 2.82E-16 |
| AL023806.1 | SPDYE1 | 0.70189866 | 2.92E-16 |
| AL023806.1 | TGM5 | 0.701837561 | 2.94E-16 |
| AL023806.1 | SLC13A4 | 0.701805475 | 2.95E-16 |
| TMEM78 | OPHN1 | 0.70177053 | 2.97E-16 |
| C8orf86 | OR52I1 | 0.701753161 | 2.97E-16 |
| TMEM78 | C2orf83 | 0.701685212 | 3.00E-16 |
| AL023806.1 | POU5F1B | 0.701630751 | 3.03E-16 |
| AL023806.1 | CTD-3105H18.18 | 0.701595807 | 3.04E-16 |
| AL023806.1 | OR7G2 | 0.701584158 | 3.04E-16 |
| TMEM78 | OR1C1 | 0.701557996 | 3.06E-16 |
| TMEM78 | OR2B11 | 0.701211415 | 3.21E-16 |
| AL023806.1 | LRRC69 | 0.701153174 | 3.23E-16 |
| TMEM78 | OR7D2 | 0.701025044 | 3.29E-16 |
| TMEM78 | CASP14 | 0.700955154 | 3.32E-16 |
| C8orf86 | GGT6 | 0.700751355 | 3.42E-16 |
| C8orf86 | CELA2A | 0.700584782 | 3.49E-16 |
| AL023806.1 | SH2D3A | 0.700430984 | 3.57E-16 |
| C8orf86 | KCNA7 | 0.700075719 | 3.75E-16 |
| AL023806.1 | OR8G1 | 0.700031451 | 3.77E-16 |
