# Supplementary material for: Selection of lncRNAs That Influence the Prognosis of Osteosarcoma Based on Copy Number Variation Data
Source: J Oncol. 2022 Mar 26;2022:8024979. doi: 10.1155/2022/8024979 (PMC8976607; doi:10.1155/2022/8024979)
Supplement: Supplementary Materials — Supplementary Figure 1: GO function annotation and KEGG pathway enrichment analyses. (A) The bubble plots for GO function enrichment (biological process). The color of the dot stands for the different P values, and the size of the dot reflects the number of target genes enriched in the corresponding pathway. (B) The bar diagrams for KEGG pathways. The y-axis represents the pathways, and the x-axis represents enriched gene numbers, and the color means adjusted P value. Supplementary Table 1: lncRNAs with >30% CNV alteration rate. Supplementary Table 2: expression profiles of 34 CNV-lncRNAs in TCGA database. Supplementary Table 3: cis-regulatory relationships of 23 mRNAs and 16 CNV-lncRNAs. Supplementary Table 4: results of Pearson analysis of coding genes significantly associated with CNV-lncRNAs. Supplementary Table 5: results of GO and KEGG enrichment analysis of 294 coding genes significantly associated with CNV-lncRNAs. Supplementary Table 6: clinical information of high- and low-risk groups in the training set. Supplementary Table 7: clinical information for the high- and low-risk groups in the test set. Supplementary Table 8: GO enrichment analysis of risk score-related genes. Supplementary Table 9: KEGG enrichment analysis of risk score-related genes. [file 8024979.f1.zip › 8024979.f6.pdf]

ONTOLOGY		ID	Description	GeneRatio	BgRatio	pvalue	p.adjust	qvalue
GO	BP	GO:005091	detection c	44/242	427/18670	5.42E-27	7.78E-24	7.78E-24
	BP	GO:00076(	sensory pe	45/242	454/18670	6.93E-27	7.78E-24	7.78E-24
	BP	GO:00509(	detection c	45/242	477/18670	5.62E-26	4.20E-23	4.20E-23
	MF	GO:00049٤	olfactory re	44/243	427/17696	5.47E-26	2.40E-23	2.26E-23
KEGG		hsa04740	Olfactory t	44/133	443/8105	1.42E-23	2.50E-21	2.50E-21

geneID	Count
OR8G1/OF	44
OR8G1/OF	45
OR8G1/OF	45
OR8G1/OF	44
26494/162	44
