# Supplementary material for: Selection of lncRNAs That Influence the Prognosis of Osteosarcoma Based on Copy Number Variation Data
Source: J Oncol. 2022 Mar 26;2022:8024979. doi: 10.1155/2022/8024979 (PMC8976607; doi:10.1155/2022/8024979)
Supplement: Supplementary Materials — Supplementary Figure 1: GO function annotation and KEGG pathway enrichment analyses. (A) The bubble plots for GO function enrichment (biological process). The color of the dot stands for the different P values, and the size of the dot reflects the number of target genes enriched in the corresponding pathway. (B) The bar diagrams for KEGG pathways. The y-axis represents the pathways, and the x-axis represents enriched gene numbers, and the color means adjusted P value. Supplementary Table 1: lncRNAs with >30% CNV alteration rate. Supplementary Table 2: expression profiles of 34 CNV-lncRNAs in TCGA database. Supplementary Table 3: cis-regulatory relationships of 23 mRNAs and 16 CNV-lncRNAs. Supplementary Table 4: results of Pearson analysis of coding genes significantly associated with CNV-lncRNAs. Supplementary Table 5: results of GO and KEGG enrichment analysis of 294 coding genes significantly associated with CNV-lncRNAs. Supplementary Table 6: clinical information of high- and low-risk groups in the training set. Supplementary Table 7: clinical information for the high- and low-risk groups in the test set. Supplementary Table 8: GO enrichment analysis of risk score-related genes. Supplementary Table 9: KEGG enrichment analysis of risk score-related genes. [file 8024979.f1.zip › 8024979.f7.pdf]

		Expression			
		Total (N=39)	High (N=19)	Low (N=20)	P value
Gender	Female	19 (48.7%)	10 (52.6%)	9 45.0%)	0.876
	Male	20 (51.3%)	9 (47.4%)	11 (55.0%)	
Age	⩾20	4 (10.3%)	2 (10.5%)	2 (10.0%)	1
	<20	35 (89.7%)	17 (89.5%)	18 (90.0%)	
Metastatic	Yes	30 (76.9%)	14 (73.7%)	16 (80.0%)	0.93
	No	9 (23.1%)	5 (26.3%)	4 (20.0%)	
