# Supplementary material for: Selection of lncRNAs That Influence the Prognosis of Osteosarcoma Based on Copy Number Variation Data
Source: J Oncol. 2022 Mar 26;2022:8024979. doi: 10.1155/2022/8024979 (PMC8976607; doi:10.1155/2022/8024979)
Supplement: Supplementary Materials — Supplementary Figure 1: GO function annotation and KEGG pathway enrichment analyses. (A) The bubble plots for GO function enrichment (biological process). The color of the dot stands for the different P values, and the size of the dot reflects the number of target genes enriched in the corresponding pathway. (B) The bar diagrams for KEGG pathways. The y-axis represents the pathways, and the x-axis represents enriched gene numbers, and the color means adjusted P value. Supplementary Table 1: lncRNAs with >30% CNV alteration rate. Supplementary Table 2: expression profiles of 34 CNV-lncRNAs in TCGA database. Supplementary Table 3: cis-regulatory relationships of 23 mRNAs and 16 CNV-lncRNAs. Supplementary Table 4: results of Pearson analysis of coding genes significantly associated with CNV-lncRNAs. Supplementary Table 5: results of GO and KEGG enrichment analysis of 294 coding genes significantly associated with CNV-lncRNAs. Supplementary Table 6: clinical information of high- and low-risk groups in the training set. Supplementary Table 7: clinical information for the high- and low-risk groups in the test set. Supplementary Table 8: GO enrichment analysis of risk score-related genes. Supplementary Table 9: KEGG enrichment analysis of risk score-related genes. [file 8024979.f1.zip › 8024979.f8.pdf]

		Expression			
		Total (N=38)	High (N=19)	Low (N=19)	P value
Gender	Female	16 (42.1%)	9 (47.4%)	7 (36.8%)	0.742
	Male	22 (57.9%)	10 (52.6%)	12 (63.2%)	
Age	⩾20	6 (15.8%)	2 (10.5%)	4 (21.1%)	0.656
	<20	32 (84.2%)	17 (89.5%)	15 (78.9%)	
Metastatic	Yes	26 (68.4%)	14 (73.7%)	12 (63.2%)	0.727
	No	12 (31.6%)	5 (26.3%)	7 (36.8%)	
