# Supplementary material for: Selection of lncRNAs That Influence the Prognosis of Osteosarcoma Based on Copy Number Variation Data
Source: J Oncol. 2022 Mar 26;2022:8024979. doi: 10.1155/2022/8024979 (PMC8976607; doi:10.1155/2022/8024979)
Supplement: Supplementary Materials — Supplementary Figure 1: GO function annotation and KEGG pathway enrichment analyses. (A) The bubble plots for GO function enrichment (biological process). The color of the dot stands for the different P values, and the size of the dot reflects the number of target genes enriched in the corresponding pathway. (B) The bar diagrams for KEGG pathways. The y-axis represents the pathways, and the x-axis represents enriched gene numbers, and the color means adjusted P value. Supplementary Table 1: lncRNAs with >30% CNV alteration rate. Supplementary Table 2: expression profiles of 34 CNV-lncRNAs in TCGA database. Supplementary Table 3: cis-regulatory relationships of 23 mRNAs and 16 CNV-lncRNAs. Supplementary Table 4: results of Pearson analysis of coding genes significantly associated with CNV-lncRNAs. Supplementary Table 5: results of GO and KEGG enrichment analysis of 294 coding genes significantly associated with CNV-lncRNAs. Supplementary Table 6: clinical information of high- and low-risk groups in the training set. Supplementary Table 7: clinical information for the high- and low-risk groups in the test set. Supplementary Table 8: GO enrichment analysis of risk score-related genes. Supplementary Table 9: KEGG enrichment analysis of risk score-related genes. [file 8024979.f1.zip › 8024979.f9.pdf]

ID	Descriptior	setSize	enrichmen	NES	pvalue	p.adjust	qvalues	rank
GO_OSSIFI	OSSIFICAT	373	0.369591	1.869862	1.00E-10	2.25E-07	2.02E-07	1564
GO_BONE_	BONE MIN	108	0.549739	2.39588	1.00E-10	2.25E-07	2.02E-07	1555
GO_BIOMI	BIOMINER.	155	0.519692	2.423858	1.00E-10	2.25E-07	2.02E-07	1555
GO_CELL_S	CELL SUBS	409	-0.45738	-1.62344	1.55E-09	2.61E-06	2.35E-06	3901
GO_OLFAC	OLFACTOF	375	0.31559	1.606352	6.58E-09	8.88E-06	7.97E-06	10110
GO_REGUL	REGULATI(	87	0.516905	2.225656	4.65E-08	5.23E-05	4.69E-05	1543
GO_REGUL	REGULATI(	70	0.550251	2.280244	6.42E-08	6.19E-05	5.55E-05	1520
GO_MITO(	MITOCHO	132	-0.5436	-1.82964	1.06E-07	8.94E-05	8.02E-05	5125
GO_VACU(	VACUOLAI	395	-0.43291	-1.53277	1.99E-07	0.000149	0.000134	4178
GO_MITO(	MITOCHO	157	-0.52056	-1.77446	5.00E-07	0.000337	0.000303	5125
GO_ANTIG	ANTIGEN F	223	-0.47583	-1.64982	8.72E-07	0.000535	0.00048	4705
GO_REPLA	REPLACEM	28	0.707698	2.426921	1.27E-06	0.000715	0.000641	729
GO_TERTI/	TERTIARY (	162	-0.50334	-1.71768	1.79E-06	0.000928	0.000833	3349
GO_ODON	ODONTO(	130	0.407145	1.895621	2.07E-06	0.000996	0.000893	805
GO_BONE_	BONE MOI	111	0.41342	1.820674	4.61E-06	0.002073	0.00186	1357
GO_MEMB	MEMBRAN	69	-0.58515	-1.84986	6.31E-06	0.002659	0.002385	4119
GO_CATAL	CATALYTI(	342	-0.42587	-1.50303	7.09E-06	0.002678	0.002403	5092
GO_ANTIG	ANTIGEN F	186	-0.47686	-1.63946	7.15E-06	0.002678	0.002403	4705
GO_REGUL	REGULATI(	182	0.348842	1.649229	7.71E-06	0.002737	0.002455	1691
GO_TRANS	TRANSLAT	103	-0.53342	-1.76696	8.25E-06	0.002781	0.002495	5622
GO_ODON	ODONTO(	90	0.444111	1.898343	1.08E-05	0.003469	0.003111	805
GO_HEART	HEART MC	248	0.310499	1.509038	1.19E-05	0.003659	0.003282	1156
GO_POSITI	POSITIVE F	38	0.602693	2.236172	1.33E-05	0.003825	0.003431	1520
GO_MITO(	MITOCHO	88	-0.54615	-1.77878	1.36E-05	0.003825	0.003431	6066
GO_ORGA	ORGANELI	474	-0.39674	-1.41382	1.71E-05	0.004529	0.004063	6009
GO_EMBRY	EMBRYON	61	0.496579	1.967717	1.75E-05	0.004529	0.004063	2743
GO_MUSC	MUSCLE C	346	0.286203	1.472112	1.93E-05	0.004819	0.004323	2525
GO_PHAG(	PHAGOCY	254	-0.44197	-1.53721	2.15E-05	0.00518	0.004647	4869
GO_ORGA	ORGANELI	86	-0.54184	-1.75716	2.42E-05	0.005636	0.005055	6066
GO_VESICI	VESICLE LU	322	-0.42174	-1.48285	2.89E-05	0.006499	0.00583	3092
GO_ANAPI	ANAPHASI	80	-0.54327	-1.75582	3.55E-05	0.007541	0.006764	3633
GO_TRANS	TRANSLAT	127	-0.49688	-1.67189	3.67E-05	0.007541	0.006764	5622
GO_DOPA	DOPAMIN	12	0.83789	2.283101	3.69E-05	0.007541	0.006764	329
GO_POSITI	POSITIVE F	45	0.548033	2.110005	4.49E-05	0.008912	0.007994	1520
GO_ENDO	ENDOCHO	72	0.45023	1.868097	4.81E-05	0.009279	0.008323	1352
GO_SENSC	SENSORY (	254	0.303944	1.462112	5.97E-05	0.011108	0.009965	1612
GO_PHAG(	PHAGOCY	126	-0.49325	-1.65915	6.09E-05	0.011108	0.009965	5602
GO_FICOLI	FICOLIN 1	59	-0.56224	-1.74352	6.46E-05	0.01133	0.010163	2674
GO_NCRN.	NCRNA MI	419	-0.39921	-1.41763	6.55E-05	0.01133	0.010163	4910
GO_PROTE	PROTEIN (	314	-0.41709	-1.46614	6.78E-05	0.011431	0.010254	5252
GO_BONE_	BONE DEV	211	0.318949	1.532834	7.27E-05	0.011965	0.010733	1357
GO_FICOLI	FICOLIN 1	124	-0.48803	-1.64022	7.68E-05	0.012215	0.010958	5350
GO_MUSC	MUSCLE SY	431	0.258624	1.330315	7.79E-05	0.012215	0.010958	1564
GO_NCRN.	NCRNA PR	345	-0.41023	-1.44798	8.94E-05	0.013482	0.012094	4907
GO_MACR	MACROPH	43	-0.61692	-1.82249	8.99E-05	0.013482	0.012094	3318
GO_MITO(	MITOCHO	56	-0.56579	-1.73615	9.24E-05	0.013543	0.012149	5622
GO_APOP	APOPTOTI	48	-0.58836	-1.76866	9.81E-05	0.013825	0.012401	2997
GO_ODOR	ODORANT	83	0.412863	1.767048	9.84E-05	0.013825	0.012401	7795
GO_VACU(	VACUOLAI	22	-0.70783	-1.827	0.000108	0.014471	0.012981	2268
GO_MUSC	MUSCLE FI	59	0.467927	1.834863	0.000108	0.014471	0.012981	2621
GO_REGUL	REGULATI(	24	0.654447	2.144154	0.000109	0.014471	0.012981	619
GO_OSTE(	OSTEOBLA	203	0.316067	1.533156	0.000116	0.01505	0.0135	1612
GO_REGUL	REGULATI(	53	-0.57269	-1.74215	0.000121	0.015152	0.013592	4119
GO_A_BAN	A BAND	38	0.553515	2.05371	0.000121	0.015152	0.013592	2691
GO_INTER/	INTERACTI	198	-0.44671	-1.54074	0.000125	0.01532	0.013742	6451
GO_MUSC	MUSCLE FI	39	0.543464	2.035861	0.000129	0.015542	0.013942	1144
GO_HEART	HEART PR(	280	0.28254	1.35231	0.000133	0.015759	0.014137	2672

GO_MITO(	MITOCHO	221	-0.43381	-1.50277	0.000136	0.015762	0.014139	6701
GO_CONN	CONNECT	259	0.293719	1.427495	0.000138	0.015762	0.014139	1427
GO_REGUL	REGULATI(	44	-0.60182	-1.78443	0.000141	0.015869	0.014235	4119
GO_PH_RE	PH REDUC	55	-0.56581	-1.73208	0.000159	0.017566	0.015757	2268
GO_STRIA	STRIATED	168	0.324337	1.520077	0.000164	0.017853	0.016015	2654
GO_SKELE	SKELETAL S	234	0.296216	1.45142	0.000184	0.019682	0.017656	1589
GO_MYEL(	MYELOID I	202	-0.43985	-1.51772	0.00019	0.020056	0.017991	4120
GO_EMBR	EMBRYON	35	0.541701	1.9702	0.000198	0.020577	0.018458	2743
GO_REGUL	REGULATI(	20	-0.72011	-1.82051	0.000201	0.02059	0.01847	1106
GO_SKELE	SKELETAL I	26	0.622173	2.083916	0.00021	0.021109	0.018936	1564
GO_CARTI	CARTILAGI	199	0.315551	1.536278	0.000218	0.021605	0.019381	1555
GO_TUMO	TUMOR NI	157	-0.46815	-1.59582	0.00023	0.022469	0.020156	4039
GO_REGUL	REGULATI(	37	-0.61057	-1.74052	0.000245	0.023626	0.021194	3607
GO_ANTIG	ANTIGEN F	75	-0.52796	-1.6901	0.000253	0.024037	0.021562	4095
GO_ENDO	ENDOSON	459	-0.38218	-1.36109	0.000292	0.027378	0.024559	5857
GO_PHAG(	PHAGOCY	69	-0.53509	-1.69159	0.000319	0.029458	0.026425	5786
GO_MITO(	MITOCHO	446	-0.38797	-1.38102	0.000338	0.030758	0.027591	5675
GO_POSIT	POSITIVE F	10	-0.82368	-1.79383	0.000342	0.030758	0.027591	2897
GO_AZUR(	AZUROPHI	151	-0.45434	-1.54781	0.000372	0.032438	0.029099	3000
GO_ESTAB	ESTABLISH	395	-0.38774	-1.37287	0.000375	0.032438	0.029099	5545
GO_SOMIT	SOMITE DI	88	0.400696	1.713869	0.000375	0.032438	0.029099	1729
GO_RIBON	RIBONUCL	396	-0.38821	-1.37451	0.000383	0.032737	0.029366	4777
GO_ENDO	ENDOCAR	35	0.526405	1.914568	0.000391	0.032986	0.02959	952
GO_MITO(	MITOCHO	472	-0.37857	-1.34926	0.000407	0.033656	0.030191	6017
GO_ACTIN	ACTIN ME	116	0.362927	1.642837	0.000409	0.033656	0.030191	2654
GO_MULTI	MULTICELI	56	0.437678	1.740912	0.000478	0.038824	0.034827	2367
GO_MYOF	MYOFIBRIL	66	0.409297	1.678874	0.000487	0.039098	0.035072	3010
GO_TERTI	TERTIARY (	72	-0.52518	-1.67027	0.0005	0.039687	0.035601	4900
GO_ANTIG	ANTIGEN F	96	-0.4881	-1.60216	0.000509	0.039886	0.035779	4095
GO_SKELE	SKELETAL I	43	0.50265	1.917525	0.000528	0.040946	0.03673	2367
GO_ENTRY	ENTRY INT	128	-0.45859	-1.54485	0.000544	0.041196	0.036955	4089
GO_NEGA	NEGATIVE	58	-0.5501	-1.7023	0.000547	0.041196	0.036955	4293
GO_PINO(	PINOCYTC	20	-0.69827	-1.76528	0.000551	0.041196	0.036955	3678
GO_PROTE	PROTEIN A	238	-0.41766	-1.45009	0.000556	0.041196	0.036955	5253
GO_ACTIN	ACTIN CYT	100	-0.48048	-1.58606	0.000578	0.042409	0.038042	3667
GO_PHAR	PHARYNGI	26	0.588055	1.969641	0.0006	0.043487	0.039009	591
GO_REGUL	REGULATI(	267	-0.40256	-1.40377	0.000608	0.043655	0.03916	4131
GO_INTEG	INTEGRIN	99	-0.4795	-1.57935	0.000641	0.044799	0.040187	3855
GO_DEVEL	DEVELOPN	34	0.531076	1.913551	0.000647	0.044799	0.040187	1821
GO_PROT(	PROTON T	10	-0.80776	-1.75916	0.000651	0.044799	0.040187	2268
GO_PROT(	PROTON T	10	-0.80776	-1.75916	0.000651	0.044799	0.040187	2268
GO_OTIC_	OTIC VESI(	15	0.72522	2.089977	0.000673	0.045858	0.041136	1367
GO_ATP_B	ATP BIOSY	29	-0.6287	-1.70625	0.000714	0.04816	0.043201	4429
GO_EARLY	EARLY END	336	-0.39086	-1.37646	0.000735	0.04911	0.044054	4235
GO_POSIT	POSITIVE F	81	0.386948	1.622018	0.000752	0.049707	0.044589	1520

leading_edcore_enrichment
tags=24%, 387733/3381/1758/162466/1280/1305/5745/121340/4656/344901/55512/655/2201/574
tags=30%, 387733/3381/162466/5745/55512/655/2201/5743/4958/2261/92/338773/55366/632/74
tags=28%, 387733/3381/1758/162466/56955/5745/258/55512/655/2201/9622/5743/249/4958/226
tags=36%, 3304/567/5170/83543/54518/9754/975/3674/1785/29780/5594/8829/51466/2273/5782
tags=97%, 26664/390275/390061/127062/284433/390323/219956/4991/81099/403284/128366/44
tags=28%, 387733/1758/162466/655/2201/4958/92/338773/632/4208/91461/54361/6662/650/931
tags=27%, 387733/162466/655/2201/4958/92/338773/632/4208/91461/54361/6662/650/490/7480
tags=58%, 6472/64960/63875/26164/84273/78988/10352/64928/6182/79590/55173/11222/90480
tags=35%, 3326/55207/4074/6560/389541/23274/28956/23339/57192/51284/9342/252839/72999
tags=54%, 6472/64960/63875/26164/84273/78988/10352/158234/64928/6182/79590/55173/1122
tags=44%, 829/4688/3119/832/3383/9493/4277/8655/653361/50856/375/3551/567/8722/55080/6
tags=43%, 162466/1280/1305/55512/4325/249/2261/338773/55790/4208/8817/2296
tags=36%, 7077/966/5768/3958/2352/5912/5724/5879/1536/3687/226/23601/4317/1729/6616/53
tags=18%, 2253/1758/4982/258/55512/655/9622/249/632/5251/7042/1747/340419/4038/650/931
tags=26%, 387733/162466/1280/1305/55512/4325/1690/249/3549/2261/5212/338773/4147/5579
tags=52%, 2624/375/23208/10347/2934/11031/4481/51411/23616/5468/2150/5879/55113/11314
tags=43%, 10595/5438/5976/10352/80149/25939/115752/158234/5511/54901/51202/51493/8074
tags=46%, 829/4688/3119/832/9493/4277/8655/653361/50856/375/3551/567/8722/55080/6891/3
tags=24%, 387733/162466/344901/655/2201/255743/4958/92/338773/632/7042/50964/4208/229
tags=59%, 65003/54534/51264/60488/64960/63875/5976/78988/64928/6182/79590/55173/11222
tags=20%, 2253/1758/4982/258/55512/655/9622/1747/340419/4038/650/9313/265/4487/256764/
tags=19%, 2253/7157/1280/1363/655/5950/3549/7134/7481/7042/4886/6091/653/129446/4633/6
tags=34%, 655/2201/92/338773/4208/91461/54361/650/490/7480/84059/659/5764
tags=66%, 56945/7818/84311/65003/54534/51264/60488/64960/63875/78988/64928/6182/79590
tags=48%, 285521/150209/7818/84233/6676/4833/84311/137682/3030/25994/6390/55744/2232/
tags=34%, 655/3549/23314/53820/80055/6926/134701/201164/4090/5727/7855/7403/10818/747
tags=27%, 4151/1160/6547/3760/5348/781/1410/3778/5743/255743/10345/7134/58/493829/4604
tags=43%, 147179/122618/79767/64005/4688/3561/55845/3394/5788/4615/10326/3553/3929/23
tags=65%, 56945/7818/84311/65003/54534/51264/60488/64960/63875/26164/78988/64928/6182
tags=30%, 5702/6279/7078/2352/80142/51411/3310/2992/671/93100/210/8635/5223/226/9861/4
tags=44%, 51434/4085/7311/5715/5711/5702/7316/5716/701/27338/9861/8881/5688/5690/1017/
tags=55%, 65003/54534/51264/60488/79048/64960/63875/51611/78988/64928/6182/79590/5517
tags=25%, 2861/7042/4929
tags=31%, 655/2201/92/338773/4208/91461/54361/650/265/490/7480/84059/659/5764
tags=32%, 162466/1280/1305/55512/4325/1690/249/3549/2261/5212/338773/4147/55790/4208/6
tags=19%, 57096/2253/1280/655/2201/5950/3549/2487/23554/53904/54549/125488/140469/129
tags=52%, 3920/10333/7879/7431/219972/10981/339122/51715/122618/4688/10435/84329/8162
tags=37%, 5879/3687/1729/535/11314/101/527/387/3071/83858/23218/10288/6556/55004/7226/
tags=39%, 115752/55656/158234/54901/6895/51202/23378/85395/84267/51493/7737/80746/849
tags=45%, 1855/51474/56893/27243/64960/63875/11198/6605/5976/23710/78988/5341/81631/9
tags=20%, 387733/2253/7157/162466/1280/1305/55512/4325/1690/249/3549/2261/5212/338773/
tags=52%, 10533/3837/5836/1432/5695/2799/272/2720/3728/3608/2547/11333/56888/2990/7916
tags=19%, 4151/1160/6547/3760/5348/781/1410/4656/3778/5743/22876/255743/10345/7134/58/
tags=41%, 55656/158234/54901/6895/51202/23378/85395/84267/51493/7737/80746/54522/8602
tags=49%, 10100/3624/4920/139716/3059/8772/375387/652/142/133396/146433/4318/1050/519
tags=66%, 65003/54534/51264/63875/78988/64928/6182/79590/11222/54148/55052/26589/2899
tags=46%, 3690/80208/10062/7301/5879/55113/11277/6347/391/8547/4240/718/720/5914/7305/
tags=93%, 390275/219956/442191/219477/219982/120586/338662/219447/26341/282775/4993/2
tags=41%, 535/1201/6556/245972/9114/54982/51606/10312/2896
tags=42%, 4656/58/4703/4015/4633/9172/4892/10324/6444/7273/10529/4617/7480/8557/56203/
tags=33%, 2253/1758/4982/340419/650/9313/265/4487
tags=23%, 3381/5745/121340/344901/655/2201/249/255743/3549/92/338773/55366/632/7481/40
tags=49%, 2624/5170/30817/613/3965/148022/6810/5724/2150/8832/695/11314/718/201294/867
tags=45%, 6271/1410/4633/4634/9172/10324/7273/442721/202333/56203/84033/287/9759/8736/
tags=55%, 983/8519/89870/10206/1642/3949/345611/1667/10898/5806/8563/1500/6737/79581/3
tags=38%, 7134/58/4604/4703/4633/4634/7135/4625/4620/7125/7273/7136/8557/4632/4606
tags=28%, 6271/6547/3760/5348/781/10345/7134/53826/7042/6505/4633/5997/4634/153/7135/5

tags=56%, 84134/116540/26519/10367/29796/51023/130916/4713/5428/4725/56922/10939/5694
tags=21%, 1302/1280/5745/80310/55512/655/1690/2822/3549/2261/5212/2487/4147/656/7481/6
tags=52%, 2624/5170/30817/613/3965/6810/5724/2150/8832/11314/201294/8673/2207/2242/221
tags=29%, 535/1201/527/537/84679/534/529/533/6556/55647/245972/9114/54982/51606/10312/
tags=31%, 4151/6547/3760/781/7134/4633/5997/4634/1837/7135/5144/4625/10324/5350/817/40
tags=21%, 387733/162466/1280/1305/55512/655/2201/4325/1690/249/3549/2261/5212/338773/4
tags=37%, 3516/2624/8111/133308/3635/3726/975/11025/6988/10859/27086/79705/10100/3624/
tags=34%, 6926/134701/201164/5727/7855/7403/10818/7471/80319/23242/10959/5046
tags=40%, 7454/10288/29108/6556/2213/6366/54209/968
tags=38%, 4656/7134/58/816/7135/4625/51778/2660/58529/3488
tags=24%, 1302/1280/5745/55512/655/1690/2822/3549/2261/5212/2487/4147/656/7481/653/557
tags=40%, 3551/944/3304/246330/8717/7293/10616/81858/8567/5715/9825/22954/5711/5702/40
tags=51%, 8829/50848/10458/4916/1613/396/3059/29085/397/25/1020/4908/3265/7226/2242/30
tags=48%, 653361/50856/3551/567/6891/5715/5711/5702/811/6890/5716/1536/9861/5688/5690/
tags=44%, 9375/64145/26276/93380/8938/3134/53373/9110/79643/79065/388/3115/127829/2360
tags=59%, 3134/3920/10333/7879/10981/339122/51715/84329/2889/7096/567/6891/57192/1785/
tags=45%, 51657/318/57128/65003/10202/549/54534/23417/11232/51264/133686/10131/201626/
tags=90%, 22985/972/146433/3725/56998/2242/920/81501/1435
tags=28%, 80142/671/93100/8635/80331/5834/5878/150372/527/1727/7415/6036/54472/2517/71
tags=42%, 55686/10096/2268/83540/8546/3566/583/6811/4033/55968/3837/4134/55755/27243/5
tags=27%, 7157/3549/2487/7481/22943/54361/9241/10655/134701/57534/5727/2296/56983/6664
tags=38%, 6895/51202/23378/84946/10946/85395/51340/10073/8602/51388/10713/55052/6130/2
tags=34%, 2253/655/7042/6091/653/6092/9241/6662/650/57057/4487/23462
tags=46%, 643246/285521/57103/51499/10013/9927/7161/164153/3980/137682/7532/25994/572
tags=34%, 3760/781/7134/58/10630/4604/4703/4633/4634/7135/5144/4625/5350/817/4620/7125
tags=29%, 4151/6547/7134/7135/51542/4625/7125/7136/26058/8557/9456/124093/342667/7138/
tags=48%, 58/4703/4633/4208/9172/4892/11155/10324/80206/7273/4205/10529/442721/8557/51
tags=49%, 2548/100049587/6843/258010/8655/84418/28956/23352/966/5912/5724/1536/3687/23
tags=44%, 653361/50856/3551/567/55080/6891/5715/5711/5702/811/6890/5716/10134/1536/221
tags=33%, 4151/6547/7134/7135/4625/7125/7136/8557/9456/124093/342667/7138/6769/844
tags=38%, 4340/3356/81603/3304/30835/975/7293/81559/10410/1605/1956/2934/3956/50848/39
tags=48%, 3929/9466/50856/3479/23208/10859/3586/3965/10457/50943/671/58509/5987/409/63
tags=65%, 1785/10062/857/81/7867/7376/257364/1794/29108/30844/51479/9261/558
tags=37%, 1111/9913/51616/254359/5595/6885/57673/8295/1387/10524/25942/51147/6877/5112
tags=38%, 29780/8829/7430/2934/50848/10458/4916/6464/116985/3645/1613/396/3059/29085/3
tags=31%, 2253/655/7042/653/53820/9241/2348/5727
tags=33%, 146850/2624/9580/8111/133308/3635/56339/11025/6988/10859/29102/51043/604/270
tags=39%, 3683/2530/683/3674/8829/3681/2013/3690/3687/7409/84695/1307/1281/27040/83605
tags=35%, 2253/6091/6092/22943/54361/6662/650/7855/30812/10818/7471/10252
tags=60%, 535/527/533/245972/9114/10312
tags=60%, 535/527/533/245972/9114/10312
tags=40%, 2253/1280/80184/6662/8557/7849
tags=59%, 2023/65018/84277/10165/6901/205/226/30968/7415/142/201163/5315/7114/7040/568
tags=34%, 124460/2889/112936/3756/221472/9392/9135/23237/54551/5604/9101/567/6653/8073
tags=27%, 655/2201/255743/92/338773/7042/4208/91461/54361/650/4090/6863/490/3572/6664/

43/4325/249/2822/255743/3549/4958/2261/92/338773/55366/656/632/7481/5251/7042/653/4015/
481/5251/4015/340419/4208/2719/91461/54361/6662/650/1278/490/7480/84059/659/55589/5764/
51/92/338773/55366/632/7481/5251/4015/340419/4208/2719/91461/54361/6662/650/9313/265/2
2/23022/6204/1605/7430/1956/2934/26136/966/3482/129804/9902/143903/4082/7531/5239/1126
2191/390037/341152/219477/390431/219982/126370/120586/283111/441669/119682/338662/39
3/265/490/23462/7480/84059/659/55589/5764/9475
0/84059/659/55589/5764
/54148/55052/283459/26589/64951/28998/51081/92170/29088/51649/387338/64432/54516/6497
1/57231/1174/10641/215/10410/27183/7311/3482/9681/2767/8140/10103/53/55332/6520/22196
2/60493/90480/54148/55052/283459/25821/26589/64951/28998/51081/92170/29088/51649/3873
891/30835/925/1785/83871/1174/5715/5711/22920/5867/5702/811/5641/6890/5971/5716/3108/1

5/11314/101/5646/79792/25852/53831/527/387/3071/28988/11322/8514/2495/83858/4318/6709/
3/265/4487/256764/2296/1278/3655/760
0/4208/6662/134701/4487/8817/2296/9321/51435/4880/5075/659/6781/389058/860/51141
/391/387/3071/51429/4240/257364/9146/718/8724/54784/100423062/2207/2213/8685/3684/5880
6/57176/5434/283459/441250/79979/51728/54512/23210/10248/28987/2193/26015/5432/57647/
0835/1785/1174/5715/5711/22920/5702/811/5641/6890/5716/3108/10134/10121/84516/1536/15
43/3398/91461/54361/9241/6662/4038/650/2735/4090/5727/6863/1959/63923/490/3572/6664/27
/2733/90480/54148/55052/26589/64951/28998/51081/29088/51649/64432/54516/64976/5018/54
2296/3655/760
092/4208/4634/1301/22943/9172/3398/9241/7135/6662/2348/650/6926/4625/57057/57534/5727/

/55173/11222/90480/54148/55052/26589/64951/28998/51081/29088/51649/64432/54516/64976/
4697/389203/65003/6714/54534/4723/2268/51264/4717/27235/1571/8034/170712/10131/60488/
1/4223/10371/80319/23242/10959/84976/5046
4/4703/4633/5997/4634/9172/1837/7135/5144/60675/444/4625/57057/10324/29895/5350/817/46
210/2889/3326/2624/3683/2214/23208/10347/10163/1785/83871/5594/2934/55423/613/63916/33
/79590/55173/11222/54148/55052/26589/64951/28998/51081/92170/29088/387338/64976/54948
317/81/5834/4860/6727/79792/25852/4282/7424/2162/1727/7415/5919/54676/6036/28988/5447
5696/26271/5699/51343/5705/891/5708/25906/5704/5691/9491/7314/5709/29882/5347/11065/5
3/11222/90480/54148/55052/9567/26589/64951/28998/51081/29088/51649/64432/64976/5018/5

6662/8817/2296/9321/51435/4880/659/6781/860
0/80184/1301/259236/9241/6662/23418/57822/4487/23424/51384/7855/145258/6664/8013/7976/
2/2889/112936/653361/7096/567/6891/23208/57192/51284/1785/83871/51466/2934/6810/30849/
2207/2357/3916/3689/10312/140885/79930
44/57176/54522/8602/643376/283459/25821/441250/389421/6130/79979/57109/51728/54512/10
793/10023/9798/64928/6597/6708/6182/829/79590/55173/140775/11222/2733/832/51534/15792
/4147/632/5251/4015/55790/4208/6678/6662/650/124976/134701/9104/1101/4090/4487/8817/22
59/3614/3326/1522/3304/23406/5594/2934/7077/5702/51411/3310/2992/210/5223/226/9861/486
493829/816/4604/4703/4633/5997/4208/4634/9172/1837/7135/5144/60675/444/4625/57057/103
/25821/441250/389421/6130/79979/57109/51728/54512/10248/10528/28987/23212/90121/6154/
5/7040/920/3976/714/6688/1435/1436
8/29088/387338/64976/54948/122704/116541/64978/128308/6150/51253/65005/740/124995/649
2621/8685/5880/558/84466/717/54209
19858/390157/390148/389090/79310/284532/390191/388761/390201/219436/401994/219981/33

4618/1525/9759/9456/9254/8736/10269/9734/4624/131377

015/340419/4208/23314/3400/3398/54361/9241/55973/650/2735/4090/265/5727/63923/3572/666
73/57506/2207/2242/2213/3684/5880/136/6813/56848/3689/3162
/219537/131377/4660
339390/2519/6714/7879/91392/1949/10533/3837/4233/5817/10318/941/8106/11043/27243/598/5

144/444/157378/4625/6445/5350/817/4090/6863/6444/490/3768/7273/388591/4205/5349/7136/4

5/7818/84311/8801/3030/6390/4697/65003/54534/4723/11232/51264/4717/60488/79135/1327/64
53/4015/55790/340419/4208/23314/1301/3400/3398/389125/91461/3221/353500/9241/7227/666
.3/3684/5880/136/6813/56848/3916/3689/3162
'2896
90/7125/6444/5224/7273/388591/7136/8557/4632/5318/5608/56203/8654/6781/3270/8048/5108
147/55790/4208/23314/1301/3221/9241/6662/134701/1101/4487/8817/2296/9321/25976/145258
'4055/5971/22985/5293/4920/9935/5468/2150/1051/5153/972/7048/139716/3428/55509/3059/87

90/340419/4208/23314/1301/389125/91461/3221/353500/9241/7227/6662/650/4090/265/4487/8
055/55504/5716/2150/9966/9861/5688/7185/5690/81/943/5696/149951/5699/5705/8718/7133/85
55/55971/4192/1436
'5696/5699/5705/3135/8517/5708/5704/5691/6892/1535/9491/5709/8673/2210/2207/5689/4689/5
03/3920/7879/55686/11252/80700/6642/78992/117584/1185/79953/55652/84888/23531/27243/51
'83871/6810/30849/811/11031/6890/9230/1536/9066/535/4218/9474/527/3135/6892/1535/533/6
'60488/18/80221/9093/3305/598/6472/64960/63875/348158/26164/78988/10352/4714/1757/1096

.9/5580/8904/4125/718/3073/201294/29108/387263/8673/302/55004/203068/5476/11010/1778/2
51042/9696/80179/1861/7109/10652/474384/84946/128866/129880/79789/5294/10016/80128/104
4/472/4617/7403/8557/5075/4618/5930/7471/4223/2294
26155/57109/54512/10248/1939/10528/54496/28987/23212/27335/3326/90121/6154/51081/5764

11/55744/81892/79065/23368/79823/389203/92667/9026/4723/11232/6548/4717/1513/2101/105
'6444/7273/388591/7136/8557/4632/5318/4606/6781/88/287/3752/23327/6093/7429/7138/776/7
'6769/844
.778/56203/8048/58529/84033/88/7138/70/8736/844/7111/91807/27063/4624/84665/3688/29765
3601/6616/11314/101/53831/527/11322/8514/1535/23218/10288/6556/23250/8673/7226/2207/96
.7/9861/100507436/5688/6396/5690/9632/5696/5699/5705/3135/8517/5708/5704/5691/6892/153

965/7726/3690/7301/10581/972/23601/857/6510/858/8372/1508/4864/8547/8764/8724/6993/867
376/4795/5443/384/84868/5914/4157/2621/558/602/945/54209/140885/54

26/26053/8193/26155/3553/6591/1743/2624/3066/54107/84243/23558/8607/79844/79683/55929/
387/26499/4739/397/9294/55742/25/1020/4908/3265/7226/9564/2242/50488/3055/79778/2252/2

086/3965/3624/50943/22985/5590/4920/441478/9935/2648/8651/1051/972/695/7048/84433/8469
5/9636/4739/3611/25/3675/8482/7076/9564/2207/3682/3055/7305/3684/2316/7094/9358/7070/46

348/9605/54209
3/10347/29058/5245/10938/10163/5594/8829/23295/91947/4644/27183/1956/3064/81615/25930/
'51430/7480/4880/84059/659/860/5764

/1290/55790/340419/50964/4208/23314/1301/22943/2719/3400/3398/91461/6678/6320/353500/5
/9451
56764/1278/490/23462/7480/84059/659/55589/6696/5764/9475/9451
1/83692/811/3690/79969/6222/6176/3936/9124/118/5879/6175/4267/6193/22888/857/10518/81/
0072/391211/392138/343171/219447/125962/26686/26341/81300/347168/403282/390436/28277

6/5018/54948/122704/63931/7284/6183/51001/116541/51335/28958/64978/65993/92399/5188/1
)/10670/80208/3108/51296/1889/81618/65082/118426/972/55315/84286/9066/6396/58528/10053
38/64432/54516/64976/5018/54948/400916/122704/63931/7284/6183/9238/51001/116541/51335
.0134/10121/84516/1536/1514/972/2217/9861/100507436/3797/5688/29127/6396/5690/10053/11

/1535/230/23218/10288/6556/23250/8673/55004/7226/23191/2207/126014/2357/963/1476/3684/

)/4627/84466/3689/54209/2209/9212
54794/51428/30834/5437/79897/9924/317781/387338/56339/29102/440400/11269/27161/25973/
14/972/2217/9861/100507436/3797/5688/29127/6396/5690/10053/9632/5696/1211/1173/5699/57
152/51430/7480/4880/84059/57045/659/860/55589/5764/3488/6096/5087
948/122704/63931/6183/65094/116541/23708/64978/65993/92399/128308/6150/51253/51373/65

/4487/10716/51384/2296/3643/7273/6664/388591/27250/6910/23462/7403/8557/5318/6654

5018/54948/122704/63931/6183/116541/64978/65993/92399/128308/6150/51253/51373/65005/5
53933/23464/401612/79135/1327/23530/598/6472/64960/63875/26164/84273/78988/4714/4702/1

20/4090/1910/6332/6442/7125/6444/490/5224/9499/3768/1131/7273/388591/3815/80852/442721
85/10458/8406/3953/4641/811/11031/4481/3690/51411/80208/23616/10062/5468/7301/2150/58
/122704/63931/6183/116541/64978/65993/128308/6150/51253/51373/65005/51021/740/124995/
2/5708/2517/9961/10490/10855/2153/3101/6709/5443/5580/4125/30/718/5054/1794/230/83716/
589/5694/991/9212
4948/122704/63931/7284/6183/54952/6176/116541/64978/65993/128308/10767/6150/51253/513

/84189/30812/4124/22882/8557/6101/5797/5584/84100/10818/51141/7849/5764/9423/10265/591
/4641/811/11031/6890/9230/8766/1536/9066/535/4218/101/9474/527/3135/71/6892/1535/8724/5

248/10528/28987/23212/2193/90121/53918/6154/57647/27341/55651/90353/79897/55759/84916
2/90480/54148/55052/5195/29979/57553/26589/64951/28998/23274/51081/29088/84305/51649/5
96/9321/3815/51435/4880/8600/79648/5075/659/6781/389058/860/51141
)/823/6727/79792/4282/1508/7415/28988/5708/9961/2495/3101/4318/230/83716/6993/7414/531
24/29895/5350/817/4620/4090/1910/6332/6442/7125/6444/490/5224/9499/3768/1131/3572/7273
57647/27341/55651/90353/79897/55759/84916/283742/25896/317781/387338/56339/29102/2858

75/51073/64981/51069/84769/51258/10573/84545/29074/10884/55168

8675/81341/219482/26494/390190/390144/219417/390195/120065/127066/150681/219453/3901

4/5179/51430/7480/4880/30812/57045/23560/659/63979/57665/860/6696/3336/3488/6096

272/9798/128866/9076/3383/23513/3695/4340/3356/84321/79228/81603/3304/440275/30835/97

1880/8557/4632/5318/5608/8654/10874/6781/3270/8048/4193/51086/10265/27129/1525/50507/1

4960/63875/78988/4714/4702/91689/4708/64928/6182/83943/79590/55173/55572/11222/51025/5
2/650/4090/265/57561/4487/8817/55553/9321/85477/4617/51435/7480/4880/30812/38/1116/659

5/287/9759/3752/23327/9456/124093/4608/342667/7138/776/6769/70/55151/5290/844/1113/514
3/6664/4617/28982/51435/7480/4880/5075/659/5079/1746/6781/389058/860/51141/84159/10265/
72/375387/652/1945/537/4286/142/133396/146433/22904/2200/4318/1050/2124/3725/1943/8451

317/55553/9321/85477/4617/51435/7480/4880/1116/659/864/1746/6781/860/4781/5915/9451
17/2874/5708/56928/8764/8742/5704/5691/9020/3875/8904/9491/7292/29108/3611/9447/7114/5

5694/10437/2209/3693
1715/153129/2047/3123/6885/4914/155382/1213/10244/55041/84946/9641/128866/3119/80230/2
556/8673/55647/245972/9114/11151/83547/5880/3916/10312
55/339983/2110/91689/200205/7084/2746/64928/6182/79590/55173/26073/11222/51025/60493/6

5798/2357/10577/23593/2512/10383/58485/311/10493/6813/3916/2896/968
494/59349/65018/9493/23513/5195/22820/23212/79090/2624/375/9700/1522/9903/81565/51203/

7/64794/8666/55299/27341/55651/55759/84916/317781/7536/4927/387338/8607/29102/6147/285

33/9049/25793/706/10981/9093/79135/3305/598/90639/11152/23710/285973/80179/4714/4702/8
0/7111/5142/4624/7341/487

/1482
3/3684/945/5328/3689/140885
5/9491/5709/8673/2210/2207/5689/4689/5694/10437/2209/3693

3/1803/3921/55223/10572/920/3303/2621/558/6404/3373/3916/57617/8692/3678/290/3693

78464/6872/10445/4967/55625/81926/25921/79969/5447/50943/2648/11091/55146/5187/4191/78
316/55971/5754/4627/4192/64098/1436

05/28959/9760/4860/8943/3059/8772/101/652/55795/54440/150372/537/4286/3135/387/3071/848
527/3689/7791/3678/3693/22801/83706

79896/3482/23307/10479/5867/11031/933/10457/474383/81614/2150/22906/65082/23163/2532/7

4361/9241/6662/4038/55973/650/134701/2735/4090/265/5727/6863/1959/8817/63923/2296/127

11078/28964/9351/57175/858/823/81873/6237/7171/5159/391/527/84964/387/79798/1601/2768
/390433/119749/127064/26735/138799/4993/126541/26539/219858/346517/119695/219874/838

28308/6150/23395/51253/51373/51654/65005/124454/51021/740/124995/64975/51073/708/5106
/51524/220002/80331/55823/535/54978/4647/221955/1201/1211/2629/6272/8943/1173/5878/11
/28958/64978/65993/92399/5188/128308/6150/23395/51253/51373/51654/65005/124454/51021/
277/9632/5696/1211/8943/1173/5699/9474/5705/11258/11021/3135/8517/1639/8907/5708/5704

1509/945/6813/3916/5328/3689/10312/1471/140885/1265/79930

5557/548644/57461/23019/10667/51163/55152/8653/9416/25819/79922/5439/117246/131965/15
05/11258/3135/8517/1639/8907/5708/5704/5691/6892/11004/1535/9491/3902/29108/6556/3835

005/51021/740/124995/64975/51073/64981/51069/51258/10573/84545/29074/10884/64949/1984

1021/740/124995/64975/51073/64981/51069/51258/10573/84545/29074/10884/64949/64968/649
32001/80775/2110/93058/91689/4708/1376/112724/151188/64928/92014/6182/5664/83943/795

/7136/8557/4632/5318/4606/5608/152/143872/56203/8654/10874/6781/3270/8048/10203/51086
79/7409/84286/9066/55113/10417/10552/11277/4647/1398/11314/6347/9474/391/3071/8547/113
64975/51073/64981/51069/84769/51258/10573/84545/29074/10884/64949/64968/64963/51116/5
201294/6993/29108/387263/7414/5315/240/54863/7114/4758/8566/64333/5709/302/203068/547

73/65005/51021/740/124995/64975/51073/285381/60678/64981/51069/54676/51258/80325/9271

5/7471/6096
33/9482/7454/6556/8673/55647/245972/55626/9114/11151/4689/83547/5880/311/6813/3916/10

/283742/25896/317781/387338/56339/29102/285855/8568/27161/54881/22984/25973/10360/106
7192/9342/6603/64432/54516/64976/5018/57231/54948/8289/400916/122704/22828/27183/508

5/240/5709/80301/1675/10120/3303/29099/5211/5689/1476/327/1460/10075/1509/1471
/388591/4205/3815/8013/80852/442721/7136/8557/51778/4632/5318/4606/5608/152/143872/56
55/8568/27161/54881/22984/10360/10667/115939/29777/23481/79922/65083/9045/117246/1319

1/219960/403273/10798/219487/441639/81168/390181/219957/343170/403274/79295/390168/4

/7293/81559/10189/10410/1605/27183/1956/2597/2934/3956/50848/3965/22954/7726/4916/36

00506013/10060/10371/287/60598/7220/796/9759/3752/23327/8862/785/7138/9254/776/59285/

54148/55052/1337/26589/126328/64951/4731/28998/51081/100532726/29088/9377/4720/26515/3
0/864/1746/6781/860/84159/4781

2/4624/7341/487
/5915/1112
1/56998/161882/130733/302/11006/3204/5914/7030/5196/861/7040/2207/2242/920/126014/8792

709/7186/8741/3303/7132/8792/10293/5689/2621/84817/5694/970/8877/51330/8771

284417/4615/51534/26872/93343/23555/309/124460/55207/3756/54432/221472/9135/389541/232

522/587/90480/55753/54148/55052/4350/10469/6648/7263/1743/122961/26589/23409/54949/649

5170/126003/23208/29058/9342/4927/1785/5874/30817/5080/3692/4085/4644/27183/25777/743

5855/8568/3692/10291/6204/27161/54881/22984/10360/57461/115939/2971/10658/9416/29777/2

31631/79594/2034/57600/4708/57154/131474/27166/83943/55572/27113/26073/84987/638/51025

62/6598/56655/10629/200186/84779/7917/3054/55274/8536/29801/6659/9474/8888/322/112869/

307/146433/2064/22806/2200/2124/3725/3902/7292/10288/1209/8451/56998/25/161882/130733/

9874/5987/857/9463/7251/55823/29062/1201/26088/8372/6272/8128/4924/5371/5878/926/12505

8/490/3572/6664/4617/27152/5179/51430/7480/4880/84059/30812/8600/57045/23560/659/6397

'3371/8321/8874/6187/2319/5297/7739/71/23164/960/726/402/3159/4739/10211/23396/1535/70
6/26532/390157/81318/390197/119694/341416/81061/390148/389090/343406/79310/284532/127

7/55157/64981/54938/51069/26995/84769/51258/8846/10573/84545/29074/10884/64949/55005/
86/81671/11337/9842/150372/4864/10325/527/8775/89849/1601/8907/54785/56928/10490/1085
740/124995/64975/51073/708/51067/55157/64981/54938/5442/51069/26995/84769/51258/8846/
/5691/6892/11004/1535/9491/3902/7454/10288/29108/6556/3835/5709/8673/24137/23180/160/1

2992/79033/5441/60625/10785/79039/22907/85441/154197/10212/10535/1662/55621/79066/994
/5709/8673/24137/160/10120/2210/2207/1778/6456/2213/162/5689/4689/83547/1175/5694/1509

/64968/64963/51116/327/55168

63/51116/55168
0/55173/55572/11222/27069/51025/622/54148/55052/65018/79188/10469/1337/54968/8834/370

/9475/88/287/796/9759/3752/23327/9456/6093/124093/4608/342667/7138/2852/6752/776/6769/
34/71/4240/51517/5580/23396/1535/5031/718/8724/1794/201294/7454/29108/6556/100423062/
5168
/1675/10120/5196/7040/7076/23191/1778/126014/29099/5211/3082/10577/5689/2621/1476/235

5/8846/10573/84545/1802/29074/10884/64949/1984/64968/1801/90850/64963/51116/55168

312/752/7791/3693

67/55152/115939/29777/23481/79922/65083/9045/117246/131965/152992/79033/10785/55636/7
1/63931/2934/6183/65094/23616/116541/23401/23708/84687/64978/1856/2150/65082/118/6599

203/8654/10874/2660/6781/2628/3270/8048/5058/58529/10611/10203/51086/221476/9475/3488
65/152992/79033/10785/55636/79039/23076/1662/4809/55621/65123/79066/5073/64852/9326/9

41608/390162/391189/390142/390152/219484/403278/390167/120066/219479/338674/283160/81

0/10670/6256/7301/10581/972/5091/23601/5987/857/7298/6510/7251/3838/858/8372/6950/537

79717/70/55151/5290/844/1113/1907/10269/779/5142/4624/7341/487/9424

87338/10245/64976/54948/5164/400916/84303/122704/63931/6183/196294/4696/100188893/55[illegible]

/7305/81501/3976/714/10272/4627/6688/10312/54209/1435/1436/284266

74/5045/23237/8216/23118/28956/11267/9101/567/133308/6653/10226/23339/57192/51284/103[illegible]

51/35/37/28998/51102/51081/10588/92170/29088/9054/5096/9361/4720/29920/84706/197322/3[illegible]

0/89958/3064/966/9919/1453/23299/3965/22920/4292/29109/4641/8729/8409/64689/474383/802[illegible]

3481/79922/65083/9045/117246/79033/51001/55636/79039/22907/23076/79084/24148/1662/48[illegible]

/10962/51142/27141/65018/10469/10193/6648/54968/8834/493753/23409/6721/126328/54949/6[illegible]

/51200/93624/84287/5977/79903/8260/8861/55733/6881/53615/90780/10856/201163/84681/898[illegible]

7001/11006/3204/5914/7030/4323/5196/861/7040/55365/2242/920/2213/7305/81501/3976/2621/

8/11188/66000/208/3135/56850/9179/28988/10490/23623/2064/55111/9146/10211/55654/8724/

9/57665/6781/860/55589/6696/81621/5764/3336/9451/3488

41/1264/8936/10867/83660/9656/5339/55742/5829/1072/3611/2580/7414/7408/3927/3675/7205/
069/26716/127059/219875/121364/120796/26245/390191/388761/390201/219436/81448/79541/

64968/285367/64963/51116/57505/55168
5/534/7317/112770/719/529/10211/8904/4125/23400/84219/84557/23324/7107/533/9482/55361/
10573/60528/84545/29074/10884/64949/55005/64968/285367/64963/51116/3028/57505/55168
0120/2210/2207/1778/6456/2213/162/5689/4689/83547/1175/5694/1509/6366/10437/54209/220

1/5073/64852/64425/8635/5188/9836/51106/114034/79042/5436/2091/23395/92675/124454/557
/10437/54209/2209/3693

8/26589/23409/114971/126328/64951/3756/4731/37/28998/284439/51081/51295/92170/1005327

56923/70/8736/55151/5290/844/7111/1146/1113/1907/26287/779
720/25/1445/55647/2212/5914/5296/23191/2207/11151/3055/10095/2213/7305/4689/2621/8685/

93/84766/1460/2512/84514/10075/10383/1509/58485/3481/10493/6280/2896/1265/87/83706/36

79039/23076/1662/4809/55621/65123/79066/5073/64852/9326/5188/9836/51106/114034/112970/
3/4943/92399/6598/128308/857/6602/6150/51253/51373/7251/10297/11078/65005/51021/1201/

836/51106/114034/112970/79042/23536/29894/2091/51654/55164/51531/79050/131870/57418/2

050/283162/219965/390445/219469/403277/26338/283189/219954/26339/219438/219968/21943

/9474/1508/4864/8547/8764/11334/7919/8724/6993/8408/8673/10155/1803/3921/55223/10572/

967/116541/28958/29928/64978/56901/65993/128308/51079/6150/80273/51253/51373/65005/51

47/252839/10938/5874/57231/9744/10410/27183/25777/1956/7311/81615/9896/9399/10067/842

7338/54516/64976/5018/54948/6821/5164/10449/2639/9997/122704/25973/2954/63931/4967/1

08/9230/81610/5293/5590/5871/8766/10121/22906/8832/118426/284021/695/283489/3797/6396

9/79066/6175/6193/26121/9326/6633/51121/51106/25804/6628/8663/51386/2091/6838/54552/5

720/4731/23274/51295/118424/3479/4846/637/9361/26100/4720/56181/5245/26515/10245/1785

/7703/10474/1487/64840/3976/51304/84885/10626/56848/6688/8877/266740/4101/28999

714/558/10272/4192/2175/54209/1435/284266

25849/348/9482/6455/4642/84868/666/30844/344/51479/9100/29934/23250/8673/55647/8263/3

2783/1465/4478/1803/4323/2022/3897/23767/23191/9564/2314/2242/3055/10095/3303/6522/585
401994/219981/122740/390155/338675/343173/8388/347468/135948/138804/219428/81472/3923

51479/10948/4758/23250/8408/8673/2783/302/23457/55004/1803/245972/25851/55846/7226/54

9/968/3693

94/51531/328/27037/131870/171568/25788/122402/56257/79650/9704/63935/51067/55157/9412

26/29088/10204/9377/51649/60386/133308/84823/84277/4720/10165/1353/137994/5245/26515/

3684/5880/558/60/311/4627/84466/3984/3689/717/54209/2209/929/140885/8877

71

79042/23536/29894/2091/23395/92675/51654/162989/124454/55164/51531/79050/131870/12240
740/10519/2629/8312/124995/64975/51073/9645/11337/64981/8775/7415/4130/51069/5499/832

85381/79650/102723631/92856/126789/54487/50628/4839/80789/115708/23195/1478/81875/61

7/219493/79290/283159/219473

7040/920/3303/79720/2621/558/6404/3373/3916/57617/8692/3678/290/3693

021/6391/740/124995/64975/51073/4728/293/64981/6687/5442/4716/4722/51069/4704/581/5453

92/81926/148022/10479/5867/11031/7316/10457/5912/118813/3108/9230/5662/55630/1889/876

0667/7284/10922/6183/9238/205/2108/57001/5106/79922/3712/1593/5160/116541/23597/6464/2

/149111/10053/150465/81/9368/4647/9632/23636/11314/1201/8943/22870/10749/1058/79998/8

5794/55164/79050/131870/124995/57418/708/8668/92856/26609/50628/6607/4839/23195/51065

/5018/400916/10449/1723/84303/9997/54471/6901/6341/3064/1347/9531/1267/81858/1352/659

02/245972/5119/23180/79803/2210/9114/6456/8630/11151/124997/920/29886/3996/79890/64601

504/7106/2274/5880/2316/7094/9358/977/5754/60/7070/4627/2275/54751/5329/3984/5328/3689/
09/81341/26737/403239/390154/26188/403244/341276/390084/219464/81470/219482/442186/26

76/29988/160/79803/8131/126321/11010/83985/9114/25798/89845/124997/2357/162/84141/5160

/54938/5442/5435/54487/4839/115708/6036/81875/25917/246721/51010/10556/56915/6035/8846

115286/64432/81894/10245/399512/64976/5018/376497/54948/400916/1723/84303/9997/122704

02/57418/56257/285381/79650/102723631/92856/126789/51067/55157/54938/54487/50628/4839/
1/57026/24144/51258/83743/10573/11004/3159/6709/64780/10300/84545/29074/84557/10884/57

37/25917/51010/10556/56915/9136/8846/27043/10607/60528/348180/54859/51093/705/55687/51

39/84769/51258/10452/10573/7386/10295/84545/29074/374291/10884/10105/64949/4580/593/64

6/51324/65082/5879/23163/84313/2217/51271/55315/84286/857/192683/55275/220002/7251/558

2907/51805/64978/65993/51218/4191/92399/80308/5091/128308/51106/6150/219/7298/27034/80

9941/84364/4744/537/113130/8775/8517/26958/1639/891/27040/434/81876/2153/84722/11004/8

/56949/81875/84769/6187/51010/10556/56915/9136/27043/10607/7919/10419/7375/51093/705/1

91/7531/57001/196294/54902/4696/5716/55967/2931/1869/2648/28958/29928/22906/55154/2308

1/3833/55697/57617/2209/56937/8877/4311

/64098/5216/84962/7791/3678/1265/9260/87/3693/2535/22801/91179/2191/4311
6534/390093/26496/401993/26693/390882/127068/390327/134083/127385/26494/390190/7932/2

06/83547/55317/64601/1175/84283/1509/1497/55697/56848/3916/57617/1291/10312/7805/2896/

6/4343/2237/9477/7919/60528/146857/55308/80119/9533/51093/51367/8449/28960/54913/1973/

4/6901/6341/25777/1347/63931/1267/1352/6183/8760/54977/83862/3958/83733/22/196294/5490

/80789/115708/23195/1478/81875/6187/25917/51010/10556/56915/9136/8846/27043/10607/605
7701/64949/1984/6455/1072/2580/64968/55201/146909/79142/8673/25851/5119/6456/64963/511

1367/8270/54913/113000/55272/88745/55644/84705/55720/6839/10436/9277/64963/3028/10978/

968/56993/26517/64963/51116/3028/55168/7384

323/535/29062/4218/26088/1211/10066/6272/8943/1173/5371/5878/926/11230/9842/55048/2364

0273/57107/23395/644096/51253/51373/2806/65005/55210/124454/55794/51021/50/54704/7282

379/10300/55143/2669/201294/7314/6993/3835/388552/199953/55201/10948/8195/79003/1020/

10856/51367/8665/79760/54913/113000/55272/8451/88745/27339/55720/8175/3921/6631/6839/1

2/51106/51079/808/80273/644096/3954/11040/55823/6834/54978/55210/409/1890/858/7332/26

82770/254786/390144/343169/119687/219417/284521/4992/26248/390195/128367/120065/1388

968/290/79156

/138428/79587/9785/5430/10436/9716/2021/10849/6039/23293/57505/23020/6499/54555/24140/

2/4696/221496/55967/1593/116541/54675/28958/51805/29928/2010/64978/56901/9941/51660/6

28/146857/348180/54859/51093/705/55687/51367/8270/54913/113000/55272/88745/55644/3265/
16/64601/327/55168/9948/58526/11344/2535/822

113179/27079/55695/81605/57505/26168/54555/24140/55505/23587/2068/26173/81887/10775

3/66000/4864/11021/527/537/3135/56850/9727/56928/84679/10490/7317/10454/113178/2064/2

94/740/10587/124995/64975/3155/51073/292/708/4728/51067/55157/64981/4357/54938/5442/4

8673/8263/5119/7462/10120/5347/2207/51693/2242/1778/55330/3996/1001/5880/553115/136/5

0436/9277/64963/51116/10978/27079/55695/23020/26168/54555/55505/10514/2068/81887/107

29/7755/3054/113115/3155/89941/292/11337/125170/4728/9474/293/28976/3002/7533/10055/3

)5/390054/283093/390036/387748/127066/390326/150681/401666/138881/26219/79544/219429/[illegible]

[illegible]10775/10594

5993/114789/3429/128308/51079/6150/7298/80273/51253/51373/2806/3954/6834/65005/55210/5[illegible]

/84705/55720/6839/79587/10436/9277/64963/57510/3028/10978/113179/27079/55695/81605/575[illegible]

3207/80772/392/9146/23396/376267/3654/23400/8724/25849/533/9482/6293/6455/7314/666/655

285/4722/51069/27349/891/4704/26995/3420/23474/54936/84769/80347/51258/25828/1892/402/

3485/3833/4627/6813/56848/3916/79083/83696/3162/4628/1294/81620/1759

75/10594/728689

0968/6687/8878/5442/4285/4716/83667/4722/142/4704/581/54539/578/57143/10452/885/25915/

283365/128368/219453/390059/8390/390151/219960/441308/442361/256144/256892/81797/4032

51021/6391/1678/740/124995/64975/51073/23761/256979/292/4728/9673/293/28976/80024/6498

505/26168/54555/24140/55505/23587/2068/26173/81887/10775/150763

6/30844/51479/10948/8673/302/55004/245972/5119/160/79803/2210/9114/8630/124997/79720/

10573/60528/10295/5095/7156/84545/3418/29074/211/10873/374291/10884/10105/64949/593/3

7249/83858/4318/7156/80119/84902/1050/23400/211/201163/84557/3725/374291/10105/203245

273/10798/219487/119679/441639/390174/81282/391196/286365/81469/127608/81309/81168/39[illegible]

31/30968/6687/10227/150290/4716/4722/51069/4704/54539/84769/51629/1349/51258/10452/57[illegible]

29886/81501/3996/4689/83547/55317/64601/85377/1175/1509/30846/55697/3916/57617/10312/[illegible]

84/64968/81889/7923/285367/8659/3421/79587/1632/217/64963/51116/3028/5888/55168/25902/

/6993/23130/29108/666/11331/10953/56993/8314/1020/2189/26517/80025/64423/80207/55626/[illegible]

0181/219957/121129/343170/122742/79317/219986/403274/120787/286362/441933/196335/3902

017/25915/11270/83858/10573/7386/11194/84545/9131/29074/374291/10884/10105/284723/6494

2209/929/968/56937/8877/79156

/2356/51205/2876

7027/23277/124997/79778/3303/3082/284114/3028/51537/5880/23593/10075/10493/4000/5329/2

261/26246/26658/124538/390437/138803/401667/158131/79295/347169/79345/390648/390168/4

9/666/5498/64968/11331/199953/23787/26517/6576/83985/2021/51117/64963/1384/8402/51116

:876/54209/2139/1759

41608/119774/390260/390162/135941/255725/26538/392392/391190/343172/391189/401992/403

/23788/51537/123096/7351/55168/7384/9016/2356/56848

[illegible]253/254879/390082/390142/135946/81696/219958/390152/341799/393046/219431/219484/3905

38/403278/390167/390892/26740/143502/390649/285659/127074/219870/402317/219983/12006[illegible]

6/79490/219479/442194/8590/81466/390265/390199/127077/390066/26333/391191/121130/3386

74/283160/26682/340980/81050/440153/81127/283162/401665/219965/390445/219952/390063/3

390883/128360/79501/219469/119678/392390/403277/219959/81328/26338/283189/390058/2199

54/26692/26339/219438/219968/138802/119692/401663/391112/283694/284383/390321/390079/

/219432/219873/125963/56656/392376/283297/125958/81442/219437/26189/219493/23538/3911

09/120775/390442/26659/391195/119764/128371/119765/390064/79324/283092/346525/119772/

/390264/26689/121275/138882/79290/283159/254783/338755/219473/391194/26476/343563/138

883/26533/341418/390077/391114/219869/390067/391192/390439/122748/127623/26497/26529/

/8392/26696/256148/8383/390038/391107/79549/341568/79334/390113/135924/130075/338751/[illegible]

144124/254973/79346/390075/120776/26531/26707/120793/346528/441911/282763/26648/81697

[illegible]/79339/81392/401427/392391/442184/162998/8387/390078/390081/441670/26493/144125/7947[illegible]

)/402135/81285
